# Rapid and Label-Free Structural Proteomics Using One-Step Swift Trypsin LiP-MS

**DOI:** 10.1021/acsomega.5c11109

**Published:** 2025-12-25

**Authors:** Yasuomi Miyashita, Ryo Konno, Satoshi Ogasawara, Yusei Okuda, Yuuki Takamuku, Toshio Moriya, Tetsuichiro Saito, Takeshi Murata, Osamu Ohara, Yusuke Kawashima

**Affiliations:** 1 Department of Applied Genomics, 34833 Kazusa DNA Research Institute, 2-6-7 Kazusa-kamatari, Kisarazu, Chiba 292-0818, Japan; 2 Department of Developmental Biology, Graduate School of Medicine, 12737 Chiba University, 1-8-1 Inohana, Chuo, Chiba 260-8670, Japan; 3 Department of Chemistry, Graduate School of Science, 12737 Chiba University, 1-33 Yayoi-cho, Inage, Chiba 263-8522, Japan; 4 Center of Quantum Life Science for Structural Therapeutics (cQUEST), 12737 Chiba University, 1-33 Yayoi-cho, Inage, Chiba 263-8522, Japan; 5 Department of Physics, School of Science, Kitasato University, 1-15-1 Kitasato, Minami-ku, Sagamihara-shi, Kanagawa 252-0373, Japan; 6 Structural Biology Research Center, Institute of Materials Structure Science, High Energy Accelerator Research Organization (KEK), 1-1 Oho, Tsukuba, Ibaraki 305-0801, Japan

## Abstract

Limited proteolysis mass spectrometry (LiP-MS) is a powerful approach for probing protein conformational changes on a proteome-wide scale. However, conventional workflows rely on a two-step digestion with proteinase K and trypsin, which increases complexity and reduces reproducibility and sensitivity. This study aimed to develop a simplified one-step protocol, termed Swift Trypsin LiP-MS (STLiP-MS), which uses a trypsin-immobilized spin column and high-speed centrifugation to achieve rapid and reproducible surface-limited proteolysis. Using HEK293 cell extracts, STLiP-MS identified 286 proteins exhibiting conformational changes upon phosphatase inhibition, including 37 enriched in phosphatase-related Gene Ontology categories. The method improvements, including suppression of predigestion and immediate enzyme inactivation, further increased sensitivity, enabling the detection of 799 proteins with structural alterations, of which 77 were enriched in phosphatase-related categories. Comparison with the single-pot solid-phase-enhanced sample preparation (SP3) method confirmed that these changes originated from structure-selective proteolysis and were not detectable under fully denaturing conditions. To demonstrate its broader applicability, we applied STLiP-MS to the adenosine A_2A_ receptor (A_2A_-BRIL) and observed antibody-induced protection of extracellular loop 2 (residues 147–176). Cryogenic electron microscopy validated Fab fragment binding to the same region, confirming the correspondence between STLiP-MS signals and actual antibody–antigen interfaces. Collectively, these results show that STLiP-MS is a rapid and robust platform that enables sensitive, label-free detection of local structural changes under near-physiological conditions and accurate prediction of protein–protein interaction sites. This method holds great promise for applications in structural proteomics and drug target identification.

## Graphical abstract

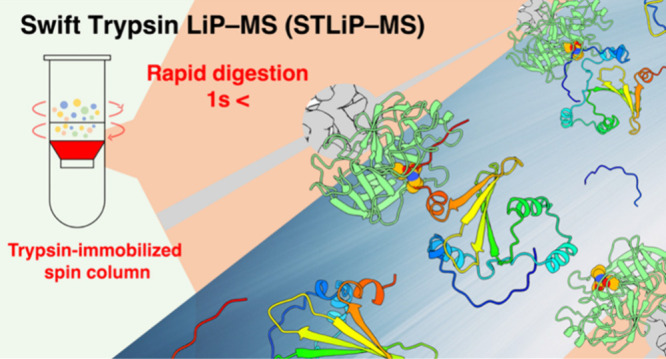

## Introduction

Protein function within cells is determined by its expression, post-translational modifications, and spatial and dynamic features such as three-dimensional structures and complex formation. Therefore, structural information is indispensable for understanding functional regulation, signal transduction, and drug responses. Recent advances in mass spectrometry (MS) have enabled proteome-wide quantification of protein abundance;^1,2^ however, methods for visualizing structural changes and conformational differences on a comparable scale remain limited.^3,4^

To address these challenges, several MS-based methods have been developed, including hydrogen–deuterium exchange MS (HDX-MS)^5,6^ and chemical cross-linking MS (XL-MS).^7,8^ HDX-MS monitors the exchange of backbone amide hydrogens with solvent deuterium, providing insights into structural flexibility and solvent accessibility. Although effective for purified protein analysis, its application to complex mixtures is limited by the reversibility of the reaction and labor-intensive sample preparation. Conversely, XL-MS investigates three-dimensional structures and complex formation by covalently linking proximal residues using cross-linking reagents, but it requires extensive optimization, generates complex data sets, and has limited throughput. Thus, neither method is sufficient for obtaining label-free, proteome-wide structural information under conditions closely approximating the native *in vivo* environment, highlighting the need for more versatile analytical strategies.

Against this background, limited proteolysis-MS (LiP-MS), originally introduced by Feng et al. and later formalized as a standardized protocol by Schopper et al., has emerged as a promising approach in structural proteomics.^9,10^ LiP-MS applies limited proteolysis to proteins in their native state, followed by MS-based identification and quantification of the resulting peptides to indirectly detect structural changes. As the protease cleavage-site accessibility is highly dependent on protein conformation, the resulting peptide patterns can differ markedly within the same sequence depending on conformational states, ligand binding, allosteric regulation, complex formation, or disease-associated mutations. A key advantage of LiP-MS is its ability to simultaneously provide structural information on thousands of proteins under label-free and nondenaturing conditions. This feature, which is difficult to achieve with HDX-MS or XL-MS, is directly applicable to complex biological samples that closely mimic the *in vivo* environment, including cell lysates, tissue extracts, and serum. Furthermore, Feng et al. demonstrated that LiP-MS can comprehensively detect structural changes within complex cell extracts.^9,10^ Since then, LiP-MS has been applied in diverse contexts, including drug target identification,^11^ metabolite–protein interaction analysis,^12^ and allosteric regulation detection.^13^ For example, Piazza et al. showed that LiP-MS-based chemical proteomics enables proteome-wide identification of metabolite–protein interactions in native cellular environments.^13^

Nevertheless, conventional LiP-MS requires a two-step proteolytic protocol comprising structure-dependent limited digestion by proteinase K, followed by complete digestion with trypsin. This procedure presents challenges in operational complexity and reproducibility, particularly during the limited digestion step, where subtle variations in enzyme concentration or reaction time can substantially alter digestion patterns, leading to overdigestion or intersample variability that compromises quantitative accuracy. These limitations underscore the need for a simplified strategy to perform LiP-MS without the constraints of complex two-step enzymatic processing.

To overcome these limitations, we aimed to develop a surface-targeted limited proteolysis method by considerably shortening the trypsin digestion reaction time, thereby enabling proteome-wide analysis of surface structural changes. Here, we report the development of an immobilized spin column-based method, termed Swift Trypsin LiP-MS (STLiP-MS), and demonstrate its proof-of-concept applications.

## Experimental Procedures

### Protein Extraction from HEK293T Cells

HEK293T cells were cultured in 10 cm dishes to 80% confluence in Dulbecco’s Modified Eagle’s Medium (Fujifilm Wako, Japan) supplemented with 10% fetal bovine serum (Thermo Fisher Scientific) and 1% penicillin/streptomycin (Fujifilm Wako) at 37 °C in a 5% CO_2_ incubator. For the STLiP-MS method, proteins were extracted from HEK293T cells by sonication for 10 min using a Bioruptor II (Cosmo Bio) in a buffer containing 100 mM Tris–HCl (pH 8.0), 20 mM NaCl, 0.5% lauryl maltose neopentyl glycol (LMNG), and 0.05% cholesteryl hemisuccinate (CHS). The lysates were then centrifuged at 12,000 × *g* for 15 min at 4 °C, and the resulting supernatants were used directly for proteolytic digestion. For the SP3 method, proteins were extracted via sonication in 100 mM Tris–HCl (pH 8.0), 20 mM NaCl, and 4% sodium dodecyl sulfate (SDS) using a Bioruptor II instrument for 10 min (30 s on/30 s off). Protein concentrations were measured using a bicinchoninic acid protein assay kit (catalog no. 23225, Thermo Fisher Scientific) and adjusted to 500 ng/μL with 100 mM Tris–HCl (pH 8.0), 20 mM NaCl, and 4% SDS.

### STLiP-MS-Based Protein Digestion and Peptide Desalting

Trypsin-immobilized spin columns (MonoSpin Trypsin, GL Sciences) were equilibrated with 50 mM ammonium bicarbonate buffer. A 0.45 μm filter (Corning, cat. no. 8162) was then placed on top of each column. Collection tubes were preloaded with 400 μL of 100% acetone prechilled to 4 °C. Subsequently, 100 μL of HEK293T cell lysate or purified protein complex (A_2A_-BRIL and antibody complexes) was loaded onto the filter and centrifuged at 10,000 × *g* for 1 min at 4 °C, enabling rapid and surface-targeted proteolysis. The eluate was collected directly into chilled acetone to quench the trypsin activity. For HEK293T cell lysates, parallel preparations were performed with and without the phosphatase inhibitor PhosSTOP (Roche) to allow for a comparative analysis. Under these conditions, undigested or high-molecular-weight proteins (such as endogenous proteases and trace trypsin) rapidly precipitate, whereas digested peptides remain in the supernatant. The acetone mixture was subjected to acetone precipitation at −25 °C for 2 h, followed by centrifugation at 12,000*g* for 5 min to remove high-molecular-weight proteins. After the supernatant was removed, the pellets were dried in a centrifugal evaporator (miVac Duo Concentrator) and resuspended in 80 μL of 50 mM Tris–HCl (pH 8.0).

For reduction and alkylation, 20 mM tris­(2-carboxyethyl)­phosphine (TCEP) was added to the solution and incubated at 80 °C for 10 min, followed by 35 mM iodoacetamide (IAA) at 27 °C for 30 min in the dark to reduce all free cysteines and convert them into a stable alkylated state. This prevents secondary disulfide bond reformation during downstream processing and ensures uniform peptide behavior in the subsequent analysis. The samples were then acidified with 20 μL of 5% trifluoroacetic acid (TFA) and desalted by using a MonoSpin C18 column (GL Sciences). The columns were prewashed with 100 μL of 80% acetonitrile (ACN) in 0.1% TFA and equilibrated with 200 μL of 3% ACN in 0.1% TFA. After sample loading, the columns were washed with 200 μL of 3% ACN in 0.1% TFA, peptides were eluted with 100 μL of 36% ACN in 0.1% TFA, and the eluates were dried in a centrifugal evaporator.

Dried peptides were reconstituted in 8 μL of 0.1% TFA and 0.01% dodecyl maltose neopentyl glycol (DMNG) and transferred to liquid chromatography–tandem mass spectrometry (LC/MS/MS) vials, and 1–2 μL was injected for analysis.

### SP3-Based Protein Digestion and Peptide Desalting

Protein digestion from cell lysates using the SP3 method was performed according to our previously reported protocol.^14,15^ First, cell lysates were reduced by adding 20 mM TCEP and incubated at 80 °C for 10 min. Alkylation was performed by adding 35 mM IAA and incubating the mixture at room temperature for 30 min in the dark.

Protein cleanup and enzymatic digestion were conducted using the SP3 method on a Maelstrom 8 Autostage (TANBead). Hydrophilic and hydrophobic Sera-Mag SpeedBeads (Cytiva) were mixed in a 1:1 (v/v) ratio and resuspended in water at a concentration of 8 μg/μL. A 20 μL aliquot of the bead suspension was added to 200 μL of the alkylated protein sample, followed by 99.5% ethanol to achieve a final concentration of 75% (v/v). The mixtures were incubated for 5 min with gentle mixing; the supernatants were removed, and the pellets were washed twice with 80% ethanol.

The beads were further resuspended in 80 μL of 50 mM Tris–HCl buffer (pH 8.0) or the same buffer containing 0.02% LMNG. Subsequently, a Trypsin/Lys-C mix (500 ng, Promega) was added, and digestion was performed overnight at 37 °C. After digestion, the samples were acidified with 5% TFA and subjected to ultrasonic treatment using a Bioruptor II (Cosmo Bio) at room temperature for 5 min.

The resulting peptides were desalted using a GL-Tip SDB (GL Sciences). For the SDB-STAGE tip, the columns were washed with 80% ACN in 0.1% TFA and equilibrated with 3% ACN in 0.1% TFA. Samples were loaded, washed with 3% ACN, and eluted with 50% or 36% ACN (each containing 0.1% TFA).

The eluates were dried using a centrifugal evaporator and reconstituted in 8 μL of 0.1% TFA or 0.01% DMNG, and a 1 μL aliquot was injected into the LC/MS/MS system.

### Data-Dependent Acquisition (DDA)-MS and Data-Independent Acquisition (DIA)-MS Using LC/MS/MS

The digested peptides were directly injected into a nanocapillary column (75 μm inner diameter × 12 cm in length; Nikkyo Technos Co., Ltd.) and maintained at 50 °C. Peptides were separated using an UltiMate 3000 RSLCnano LC system with mobile phases A (0.1% formic acid in water) and B (0.1% formic acid in 80% acetonitrile). The eluted peptides were analyzed by using an Orbitrap HF-X mass spectrometer (Thermo Fisher Scientific) equipped with an InSpIon system (AMR, Tokyo, Japan) (PMID: 37036810).

For DDA-MS analysis, peptides were eluted using a 70 min linear gradient (0 min at 8% B, 62 min at 37% B, 68 min at 75% B, and 70 min at 75% B) at a flow rate of 200 nL/min. MS1 spectra were acquired at a resolution of 60,000 over a scan range of 380–1,240 *m*/*z*, with an automatic gain control (AGC) target of 3 × 10^6^ and a maximum injection time of 100 ms. The top 50 precursor ions with charge states of 2+ to 5+ and intensities >2.0 × 10^5^ were selected for fragmentation using higher-energy collisional dissociation with stepped normalized collision energies of 22, 25, and 28%. MS2 spectra were acquired from 200 *m*/*z* at a resolution of 30,000, with an AGC target of 1 × 10^4^, and an automatic maximum injection time. The dynamic exclusion was set to 30 s.

For DIA-MS analysis, peptides were separated using a 70 min gradient (0 min at 10% B, 62 min at 37% B, 68 min at 75% B, and 70 min at 75%) at a flow rate of 200 nL/min. MS1 spectra were acquired across a range of 405–1005 *m*/*z* at a resolution of 30,000, with an AGC target of 3 × 10^6^ and a maximum injection time of 55 ms. MS2 spectra were acquired from 200 *m*/*z* at a resolution of 30,000, an AGC target of 3 × 10^6^, a maximum injection time of 60 ms, and a normalized collision energy of 23%. The isolation window for MS2 was set to 10 Th, and a variable window scheme covering the 400–1000 *m*/*z* range was generated using Xcalibur 4.3 (Thermo Fisher Scientific).

### Protein Expression

The A_2A_-BRIL construct was designed based on previously reported sequences incorporating stabilizing mutations.^16–18^ Our original construct contains an N-terminal FLAG tag (DYKDDDDK) for monitoring membrane expression, a C-terminal HRV3C protease recognition site (LEVLFQ/GP) to allow controlled elution from affinity resin, mNeonGreen for estimating expression levels, and an 8 × His tag for purification. This construct was cloned into a pEG-based expression vector. Expi293F cells (Thermo Fisher Scientific) were cultured in HE200 CD medium (Gmep Inc.) supplemented with 4 mM l-alanyl-l-glutamine and a penicillin/streptomycin/amphotericin B mixture (all from Nacalai Tesque Inc.). Cultures were maintained in 125 mL flasks containing 30–50 mL of medium, shaken at 130 rpm in a humidified incubator at 37 °C with 8% CO_2_. For transfection, 500 μg of plasmid DNA and 2.5 mL of PEI Max (1 mg/mL; Polysciences) were diluted in 25 mL of Opti-MEM (Thermo Fisher Scientific), mixed, and incubated for 15 min at room temperature. The resulting complex was added to 500 mL of cells at a density of 3 × 10^6^ cells/mL, and the culture was maintained at 130 rpm. After 24 h, valproic acid (Tokyo Chemical Industry Co., Ltd.) was added to a final concentration of 3.5 mM. Cells were harvested 48 h post-transfection by centrifugation at 3000 × *g* for 5 min, washed with phosphate-buffered saline (PBS), snap-frozen in liquid nitrogen, and stored at −80 °C.

### Flow Cytometry

Transfected cells were washed with PBS containing 0.1% bovine serum albumin and incubated with a primary anti-DDDDK-tag (anti-FLAG-tag) monoclonal antibody (MBL, Japan) or anti-A_2A_ mAb^16^ for 1 h at 4 °C. After washing, the cells were incubated with an Alexa Fluor 647-conjugated antimouse IgG secondary antibody (Jackson Immuno Research, USA) for detection. Following additional washes, fluorescence data were acquired by using a CytoFLEX flow cytometer (Beckman Coulter).

### Protein Purification and Complex Formation for IgG-Bound A_2A_-BRIL

Cell pellets obtained from a 500 mL Expi293F suspension culture were solubilized in a buffer containing 20 mM 4-(2-hydroxyethyl)-1-piperazineethanesulfonic acid (HEPES) (pH 7.4), 300 mM NaCl, 1% *n*-dodecyl-β-d-maltoside (Anatrace), and 0.2% cholesteryl hemisuccinate (CHS). The suspension was stirred for 2 h at 4 °C and ultracentrifuged at 150,000 × *g* for 45 min, and the insoluble fraction was discarded.

To isolate 8 × His-tagged A_2A_-BRIL from the soluble fraction, an anti-His-tag monoclonal antibody–immobilized resin was prepared using NHS-activated Sepharose (Cytiva, Illinois, USA). In this study, 3 mL of the anti-His antibody–immobilized resin was used, which contains approximately 3 mg of IgG/mL resin and provides an effective binding capacity of about 1.0 mg of His-tagged protein per mL. This amount was sufficient to capture the full yield of A_2A_-BRIL. The protein amount throughout the purification process was estimated using the fused fluorescent protein, mNeonGreen, as a quantitative indicator. After binding, the resin was washed with a buffer containing 0.025% LMNG. The bound protein was eluted by cleavage with HRV3C protease. The eluate was concentrated using an Amicon Ultra centrifugal filter unit (50 kDa MWCO, Merck Millipore) and stored at −80 °C. From the 500 mL Expi293F suspension culture, approximately 2 mg of purified A_2A_-BRIL was obtained.

Purified A_2A_-BRIL was mixed with an in-house–produced anti-A_2A_ IgG antibody at a 1:1.5 molar ratio and incubated overnight at 4 °C. The mixture was subjected to size exclusion chromatography (SEC) on a Superose 6 Increase column equilibrated with 20 mM HEPES (pH 7.4), 300 mM KCl, and 0.005% LMNG. Complex formation was verified from the SEC elution profile, and peak fractions corresponding to the complex were pooled and concentrated to 1 mg/mL.

### Data Analysis

DIA-MS files were searched using DIA-NN (version 1.9.2, https://github.com/vdemichev/DiaNN)^19^ against a human in silico–generated spectral library constructed from the UniProt human protein sequence database (downloaded in March 2024; 20,575 entries; UP000005640) using DIA-NN. The parameters for spectral library generation were as follows: proteolytic enzyme, trypsin; maximum of one missed cleavage; peptide length, 7–45 amino acids; precursor charge range, 2–4; precursor *m*/*z* range, 350–1250; and fragment ion *m*/*z* range, 200–1800. The following fixed and variable modifications were applied: N-terminal methionine excision and cysteine carbamidomethylation. The DIA-NN search parameters were as follows: precursor mass accuracy, 10 ppm; MS1 accuracy, 10 ppm; protein inference and gene expression were enabled; and match-between-runs were disabled. The protein identification threshold was set at an ≤1% false discovery rate (FDR) at both the precursor and protein levels. Proteins containing unique peptides were selected for downstream analyses. For cross-sample quantitative comparisons, proteins were retained if valid values were detected in at least 70% of the samples within an experimental group. Quantitative coefficients were calculated using Perseus v1.6.15.0 (https://maxquant.net/perseus/).^20^

For purified A_2A_-BRIL and A_2A_-BRIL-IgG samples, DDA-MS data were processed using PEAKS Studio 12.5 (Bioinformatics Solutions Inc.). Searches were performed against the UniProt human protein sequence database (downloaded in April 2025; 20,644 entries; UP000005640), and the A_2A_-BRIL construct sequence was obtained from the Protein Data Bank (PDB ID: 5IU4). The A_2A_-BRIL sequence was exported from the corresponding PDB entry and edited to incorporate the introduced mutation. The same FASTA file was also supplemented with the IgG Fab region sequence.

For FDR estimation, a reverse decoy database was automatically generated by PEAKS. Search parameters were as follows: proteolytic enzyme, trypsin; enzymatic specificity, semitryptic; up to four missed cleavages; peptide length, 7–50 amino acids. Cysteine carbamidomethylation and methionine oxidation were set as fixed and variable modifications, respectively. Identifications were filtered to ≤1% FDR at both the peptide and protein levels by using the PEAKS decoy-based method.

### Cryogenic Electron Microscopy (Cryo-EM) Sample Preparation and Data Acquisition for the A_2A_-BRIL-Fab Complex

Purified A_2_A-BRIL was mixed with the in-house–produced anti-A_2A_ Fab at a 1:1 molar ratio and incubated for 2 h at 4 °C. The complex was subjected to SEC using a Superose 6 Increase column equilibrated with a buffer containing 20 mM HEPES (pH 7.4), 300 mM KCl, and 0.005% LMNG. The peak fractions were collected and concentrated to 10 mg/mL.

A 3 μL aliquot of the sample was applied to a glow-discharged Quantifoil R1.2/1.3 grid using a Vitrobot Mark IV (Thermo Fisher Scientific, Oregon, USA). Grids were blotted for 5 s with a blot force of 10 at 18 °C and 100% humidity and rapidly vitrified in liquid ethane.

Cryo-EM data were collected using a Titan Krios electron microscope (Thermo Fisher Scientific) operated at 300 kV. Images were recorded using a Falcon 4i direct electron detector in electron-counting mode. A total of 50 movie frames were recorded per exposure at a dose rate of 1.0 e^–^/Å^2^ per frame. The nominal defocus range was set from −0.8 to −2.0 μm in 0.4 μm intervals. The physical pixel size was 0.75 Å.

### Cryo-EM Image Processing and Model Building

Image processing was conducted using cryoSPARC v4.6.0.^21^ Motion correction and contrast transfer function (CTF) parameter estimation were performed using Patch MotionCorr and patch CTF estimation, respectively.

A total of 9950 micrographs were obtained. Particles (7,671,814) were autopicked using the blob picker and extracted at 3.0 Å/pixel (box size: 256, binned to 64). After two-dimensional (2D) classification (200 classes, 40 iterations), 6,412,355 particles were retained for the heterogeneous refinement. A second round of 2D classification (400 classes, 40 iterations; circular mask diameter 228 Å) yielded 2,450,688 particles, which were subjected to *ab initio* reconstruction in cryoSPARC to generate initial maps. Two rounds of *ab initio* reconstruction were performed. In the first round, three classes were generated, with the maximum resolution set to 12 Å, initial resolution of 20 Å, initial minibatch size of 300, final minibatch size of 1000, and class similarity set to 0. From the resulting volumes, the class that best represented the A_2A_-BRIL-Fab complex was selected for further analysis. In the second round, parameters identical to those in the first round were used, except that two classes were generated with the maximum resolution set to 6 Å, initial resolution set to 15 Å, and class similarity set to 0.1. Subsequent heterogeneous refinement (using all *ab initio* maps) identified a high-quality class of 701,249 particles, which were re-extracted at 0.75 Å/pixel (box size 360) and refined by nonuniform refinement. Additional heterogeneous refinement (using all previous heterogeneous refinement maps) yielded 172,394 particles, which were further processed by nonuniform refinement to produce a 3.41 Å reconstruction. To enhance the local resolution, a soft mask encompassing the Fab fragment and A_2A_-BRIL region was generated and applied for local refinement, resulting in a final reconstruction at 3.45 Å resolution, as estimated by the gold-standard FSC 0.143 criterion (Figure S1).

The initial model of the A_2A_ receptor was derived from a previously reported structure (PDB: 5IU4), excluding BRIL fusion. The final cryo-EM map was first fitted by rigid-body docking in UCSF Chimera, followed by manual rebuilding–particularly of extracellular loop 2 (ECL2)–in Coot and real-space refinement in Phenix. Molecular graphics were prepared using UCSF Chimera.

## Results and Discussion

### Establishment of a Rapid Limited Proteolysis Method Using a Trypsin-Immobilized Spin Column

We developed a simplified STLiP–MS method using a trypsin-immobilized spin column (MonoSpin Trypsin) to achieve structure-dependent, limited proteolysis at the protein surface through instantaneous digestion (Figure 1). Protein solutions under native conditions were passed through the spin column by high-speed centrifugation (10,000 *× g*). Although the manufacturer’s instructions recommend gradual digestion at 200 *× g*, applying 10,000 *× g* (50-fold higher) enabled nearly instantaneous passage of the sample through the trypsin-immobilized monolithic layer. As the column elutes 0.5 mL in <10 s at 1000 × *g*, it is likely that at 10,000 × *g*, elution occurs during the initial acceleration phase, before reaching maximum speed.

**Figure 1:**
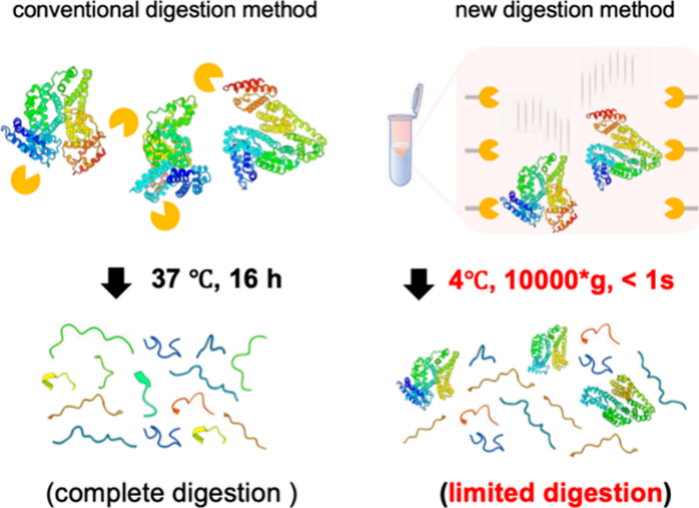
Rapid, surface-limited LiP-MS with an immobilized-trypsin spin column. Schematic comparison of the conventional digestion workflow (left) and the immobilized-trypsin method developed in this study (right). Conventional LiP-MS requires complete digestion (37 °C, 16 h), whereas the single-step immobilized-trypsin spin column (4 °C, 10,000 × *g*, <1 s) restricts cleavage to exposed regions, generating structure-dependent peptides with greater speed and reproducibility. LiP-MS, limited proteolysis-mass spectrometry.

The centrifuge used in this study exhibited a highly reproducible acceleration of 10,000 × *g*, ensuring consistency across runs. As most commercial centrifuges display comparable acceleration reproducibility, this factor is unlikely to represent a major limitation. Based on the observed flow rates at 1000 × *g*, the estimated protein–matrix contact time was <1 s. Conversely, such rapid proteolysis cannot be achieved with conventional in-solution digestion, where addition, mixing, and quenching require longer periods. Thus, the combination of a trypsin-immobilized spin column with high-speed centrifugation enables this novel limited proteolysis method.

To assess the utility of STLiP-MS, we examined structural changes in HEK293 cell extracts with or without the phosphatase inhibitor. Following limited proteolysis, high-molecular-weight proteins were removed via acetone precipitation, peptides were purified using a C18 column, and the samples were analyzed using LC/MS/MS (Figure 2A). As a result, 286 proteins exhibiting structural changes were identified (Figure 2B and Table S2). Gene Ontology (GO) analysis revealed significant enrichment of phosphorylation-related categories, including “phosphatase activity” (GO:0016791), “protein tyrosine phosphatase activity” (GO:0004725), and “serine/threonine phosphatase activity” (GO:0004722) (Figure 2C). Among these, 37 proteins likely underwent phosphatase inhibitor–induced structural alterations, demonstrating that this method can effectively detect phosphorylation-dependent conformational changes.

**Figure 2:**
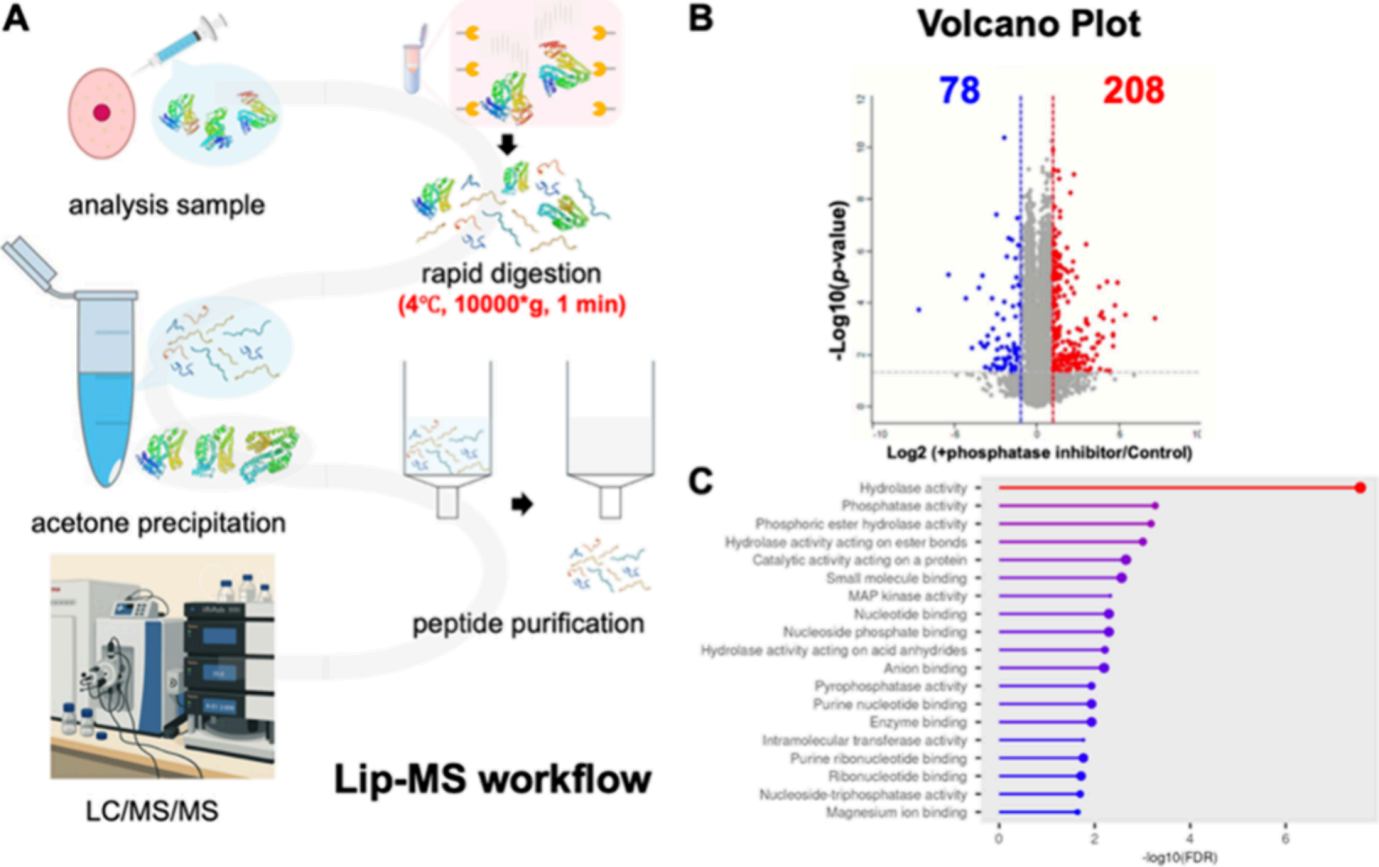
STLiP-MS workflow and structural changes in HEK293 lysates induced by phosphatase inhibition. (A) Schematic of the single-step, STLiP-MS work; flow. Native cell lysates are digested in a single pass through an immobilized-trypsin spin column (4 °C, 10,000 × *g*, 1 min), followed by acetone precipitation, peptide cleanup, and LC/MS/MS. (B) Volcano plot of protein-level LiP changes between + phosphatase inhibitor and control. Axes: *x*, log2­(+phosphatase inhibitor/control); *y*, −log10­(*p* value). Dashed lines indicate fold change and significance cutoffs. In total, 286 proteins were observed to be significant, of which 208 were increased and 78 were decreased in susceptibility (red and blue, respectively). (C) Gene Ontology enrichment analysis of the 286 proteins. Phosphatase-related terms are enriched (such as nucleoside-triphosphatase regulator activity). The *x*-axis shows −log10­(FDR). STLiP-MS, Swift Trypsin limited proteolysis–mass spectrometry; LC/MS.MS, liquid chromatography-tandem mass spectrometry; LiP-MS, limited proteolysis–mass spectrometry.

### Improvement of the STLiP-MS Protocol Enhances Sensitivity for Detecting Structural Changes

To further refine the method, we optimized both the control of the limited digestion reaction and the protease-inactivation step. In conventional protocols, uncontrolled predigestion can occur due to unintended contact between the sample and immobilized trypsin before centrifugation. To address this issue, we introduced a filter placed above the column to physically block contact until the reaction was centrifuged (Figure 3A). With this modification, the number of proteins exhibiting structural changes increased to 332, with 44 proteins significantly enriched in the phosphatase-related GO categories (Figure 3B,C).

**Figure 3:**
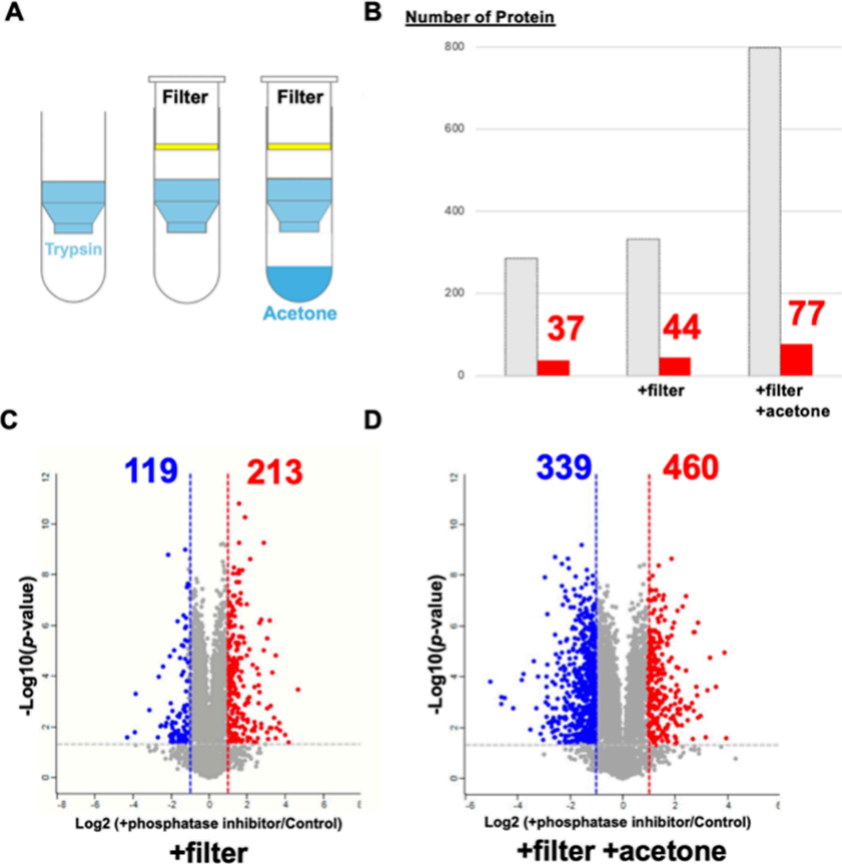
Upper filter and immediate acetone quench enhance STLiP-MS sensitivity. (A) Schematic illustration of two modifications to the immobilized-trypsin spin column protocol: placing a filter above the enzyme bed to prevent unintended contact before centrifugation (thereby suppressing predigestion) and collecting the eluate directly into acetone to rapidly inactivate residual trypsin and endogenous proteases. (B) Number of proteins showing significant LiP changes in HEK293 lysates (+phosphatase inhibitor vs control): gray, total; red, phosphatase-related GO terms. Counts increased from 286 (filter) to 799 (filter + acetone); phosphatase-related proteins increased from 37 to 77. (C, D) Volcano plots of protein-level LiP changes for the two optimized conditions (+phosphatase inhibitor vs control). Axes: *x*, log2­(+phosphatase inhibitor/control); *y*, −log10­(*p* value). Dashed lines indicate fold change and significance cutoffs. With the filter alone (C), 332 proteins were significant: 213 increased and 119 decreased in susceptibility (red and blue, respectively). With the filter + acetone quench (D), 799 proteins were significant: 460 increased and 339 decreased. STLiP-MS, Swift Trypsin-limited proteolysis–mass spectrometry; LiP-MS, limited proteolysis–mass spectrometry; GO, Gene Ontology.

To further prevent trypsin leakage from the spin column and suppress overdigestion by endogenous proteases in the cell extracts, the effluent was immediately collected in acetone after elution, enabling rapid enzyme inactivation (Figure 3A). This modification markedly enhanced the sensitivity and specificity of structural change detection, ultimately identifying 799 candidate proteins with altered structures. Among these, 77 proteins were significantly enriched in phosphatase-related GO categories (Figure 3B,D and Table S3).

These findings demonstrate that even minor refinements to the one-step LiP-MS protocol using trypsin-immobilized columns can substantially improve the sensitivity to detecting structural changes. Suppressing predigestion and immediately inactivating proteases proved highly effective in improving reproducibility and accuracy in quantitative comparisons of structural changes and are expected to contribute to the standardization of the experimental system.

### Specificity Evaluation of STLiP-MS by Comparison with the SP3 Method

Using this protocol, we identified 799 proteins that exhibited structural changes in response to the phosphatase inhibitor, of which 77 were significantly enriched in phosphorylation-related GO categories (Figure 3D). To evaluate whether these changes specifically reflect structure-dependent limited proteolysis, we compared the results with those obtained using the SP3 method (Single-Pot Solid-Phase–enhanced Sample Preparation), a conventional proteomics workflow based on complete digestion.^14,15^ In the SP3 protocol, proteins are fully denatured in SDS-containing buffer, purified using magnetic beads, and digested with trypsin for 16 h at 37 °C. Under these conditions, structural information is lost and the resulting peptide profiles primarily reflect linear amino acid sequences.

When the phosphatase inhibitor was applied to the same samples using the SP3 method and analyzed by LC-MS/MS, only 25 proteins showed significant changes with no enrichment detected in GO analysis (Figure 4A,B). This indicates that phosphatase inhibitor–induced structural changes are difficult to detect under fully denaturing conditions but become apparent only under structure-selective conditions, such as those achieved with STLiP-MS (Figure 4C). Thus, STLiP-MS, which is based on structure-selective cleavage, enables the detection of structural and functional protein alterations that cannot be captured by conventional complete digestion protocols such as SP3.

**Figure 4:**
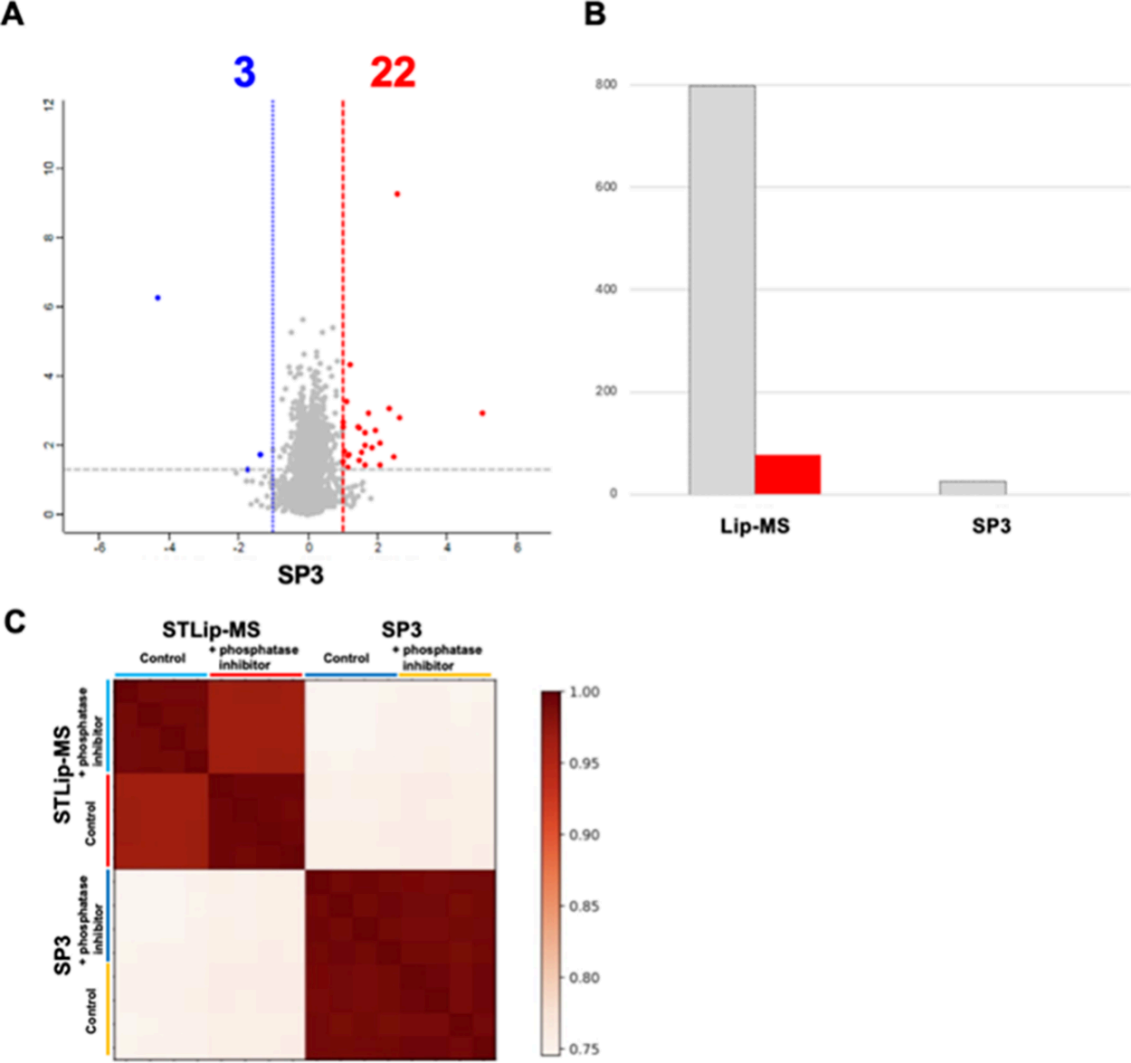
STLiP-MS-derived surface-limited digestion reveals phosphatase inhibitor effects missed by SP3. (A) Volcano plot for the SP3 (complete digestion) workflow comparing + phosphatase inhibitor vs control. Axes: *x*, log2­(+phosphatase inhibitor/control); *y*, −log10­(*p* value). Dashed lines indicate the fold change and significance cutoffs. Only 25 proteins were observed to be significant, of which 22 were increased and 3 were decreased in susceptibility (red and blue, respectively). (B) Number of significant proteins detected using STLiP-MS vs SP3. Gray, total significant proteins; red, proteins annotated with phosphatase-related GO terms. STLiP-MS: 799 total, 77 phosphatase-related; SP3:25 total, 0 phosphatase-related. (C) Sample-wise correlation heatmap across the methods and conditions. In STLiP-MS, + phosphatase inhibitor and control samples were separated, whereas SP3 profiles appeared more similar between conditions, consistent with reduced sensitivity under denaturing, complete-digestion conditions. STLiP-MS, Swift Trypsin-limited proteolysis–mass spectrometry.

### Prediction of Antibody Binding Sites Using STLiP-MS

These results demonstrate that STLiP-MS, through structure-selective cleavage, can capture structural and functional alterations that cannot be detected by conventional full-digestion protocols, such as SP3. We next evaluated whether STLiP-MS can be applied to protein complexes to infer their interaction interfaces. As a model system, we analyzed the complex formed between the adenosine A_2A_ receptor and its conformation-specific antibody. A_2A_R, a member of the G-protein-coupled receptor (GPCR) family, binds adenosine as its ligand and regulates diverse physiological processes. It is widely recognized as an important therapeutic target across multiple disease areas. We generated an antibody that specifically recognizes the native three-dimensional structure of A_2A_R,^16^ but its binding epitope has not yet been identified. As this conformation-specific antibody stabilizes the extracellular structure of A_2A_R and serves as a valuable tool for structural and functional studies, elucidating its binding site is essential for understanding the structural basis of antibody-mediated stabilization. Furthermore, like many GPCRs, A_2A_R is inherently unstable as a membrane protein; therefore, a stabilized A_2A_-BRIL fusion construct has been widely used to facilitate structural analysis and complex formation.^16–18^ In this study, we used the A_2A_-BRIL-antibody complex as a model system to examine whether STLiP-MS can identify the antibody-binding region.

A stabilized A_2A_-BRIL construct^16–18^ was expressed, purified, and incubated with IgG antibodies, and complex formation was confirmed using SEC and SDS-polyacrylamide gel electrophoresis (PAGE) (Figure 5A).^22–25^ STLiP-MS was subsequently applied to both A_2A_-BRIL alone and the antibody complex, and the resulting peptide profiles were compared. Several peptide regions exhibited altered signal intensities (Figures S2), suggesting structural alterations upon antibody binding. Residues 147–160 (MLGWNNCGQPKEGK) and 161–176 (QHSQGCGEGQVACLFE), corresponding to ECL2, showed markedly reduced cleavage susceptibility in the antibody complex, indicating that these regions are likely positioned near the antibody-binding site (Figure 5B,C).

**Figure 5:**
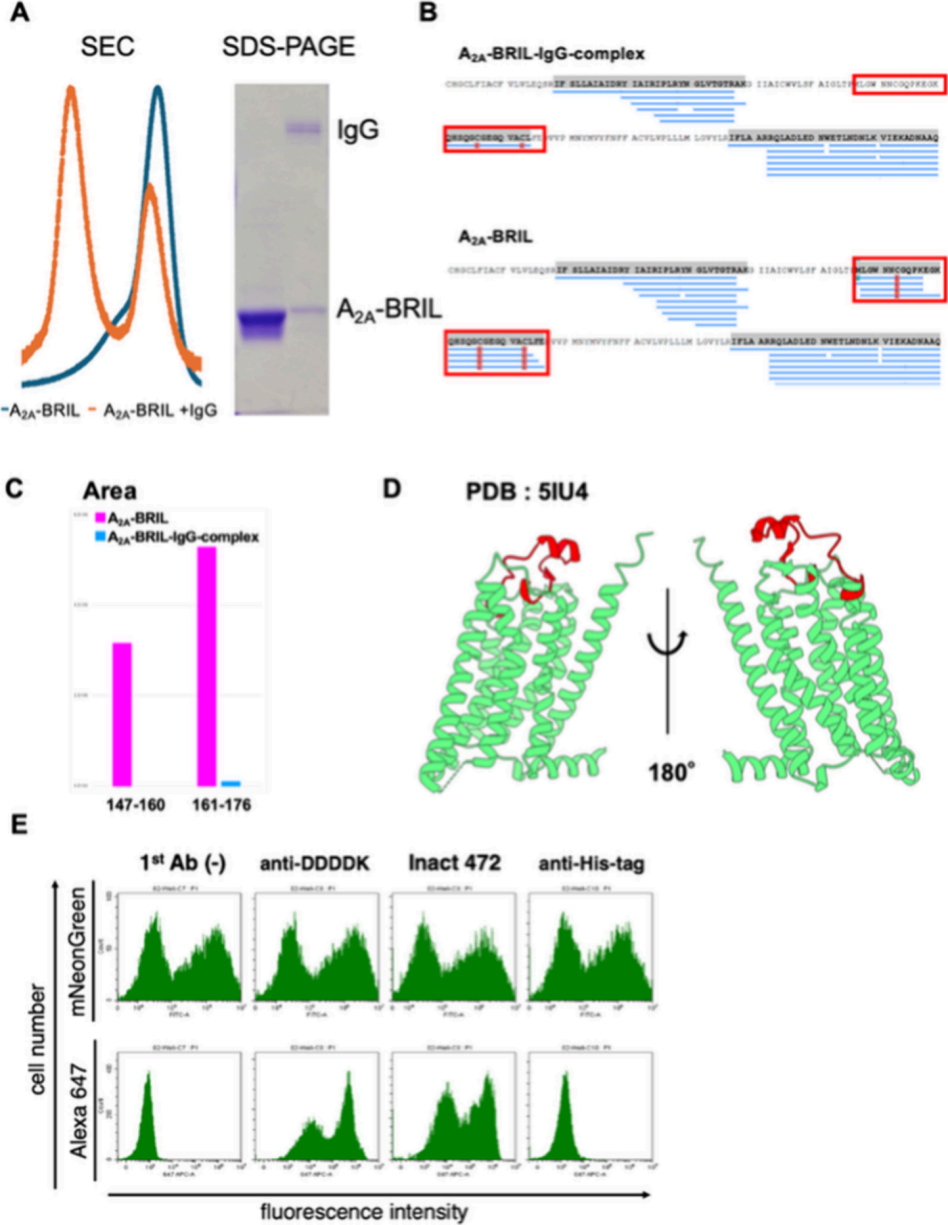
STLiP-MS reveals the antibody-binding surface on A_2A_-BRIL via protection from proteolysis. (A) SEC chromatograms and SDS-PAGE confirming the formation of the A_2A_-BRIL-IgG complex. (B) STLiP-MS peptide-coverage maps for A_2A_-BRIL alone and A_2A_-BRIL-IgG complex. Peptides whose abundance decreases upon complex formation (consistent with protection from proteolysis) are boxed in red; notably, residues 147–160 (MLGWNNCGQPKEGK) and 161–176 (QHSQGCGEGQVACLFE). (C) Extracted-ion peak areas for peptides 147–160 (magenta) and 161–176 (blue) are markedly reduced in the A_2A_-BRIL-IgG complex relative to A_2A_-BRIL alone, indicating protection by antibody binding. (D) Mapping these peptides onto the A_2A_ receptor structure (PDB 5IU4) places them in ECL2 (red); two views rotated by 180° are shown, consistent with the extracellular epitope. (E) Flow cytometry validation of antibody binding to A_2A_-BRIL. HEK293 cells transiently expressing A_2A_-BRIL-mNeonGreen were incubated with the indicated primary antibodies (no first antibody [first Ab (−)], anti-DDDDK/FLAG, Inact 472, and anti-His-tag) followed by an Alexa Fluor 647–conjugated secondary antibody. Upper histograms: mNeonGreen fluorescence (expression control). Lower histograms: Alexa 647 fluorescence report antibody binding. The first Ab (−) condition served as a secondary-only control to assess the background. STLiP-MS, Swift Trypsin limited proteolysis-mass spectrometry; SEC, size exclusion chromatography; SDS-PAGE, sodium dodecyl.

Mapping these peptides onto a reported structure (PDB ID: 5IU4) confirmed that both regions were located within extracellular loops, consistent with structural protection upon antibody binding (Figure 5D).^25^ Flow cytometry further verified binding of the antibody to the extracellular domain (Figure 5E).

Overall, these results demonstrate that STLiP-MS using trypsin-immobilized spin columns can sensitively detect local structural changes induced by antibody binding at the peptide level, thereby providing a powerful approach to infer antibody–antigen interaction sites.

### Structural Analysis of Antibody Complexes by Cryo-EM

To confirm whether the structural regions of the A_2A_ receptor detected using STLiP-MS correspond to the antibody-binding sites, we performed cryo-EM analysis of the receptor–antibody complex. As intact IgG antibodies (∼150 kDa) are large and hinder particle alignment and three-dimensional reconstruction in single-particle analysis, Fab fragments (∼50 kDa) containing variable regions were generated by enzymatic cleavage to obtain molecules suitable for structural analysis. Purified A_2A_-BRIL was incubated with Fab fragments, and the complex formation was confirmed under optimized conditions using SEC and SDS-PAGE (Figure 6A).

**Figure 6:**
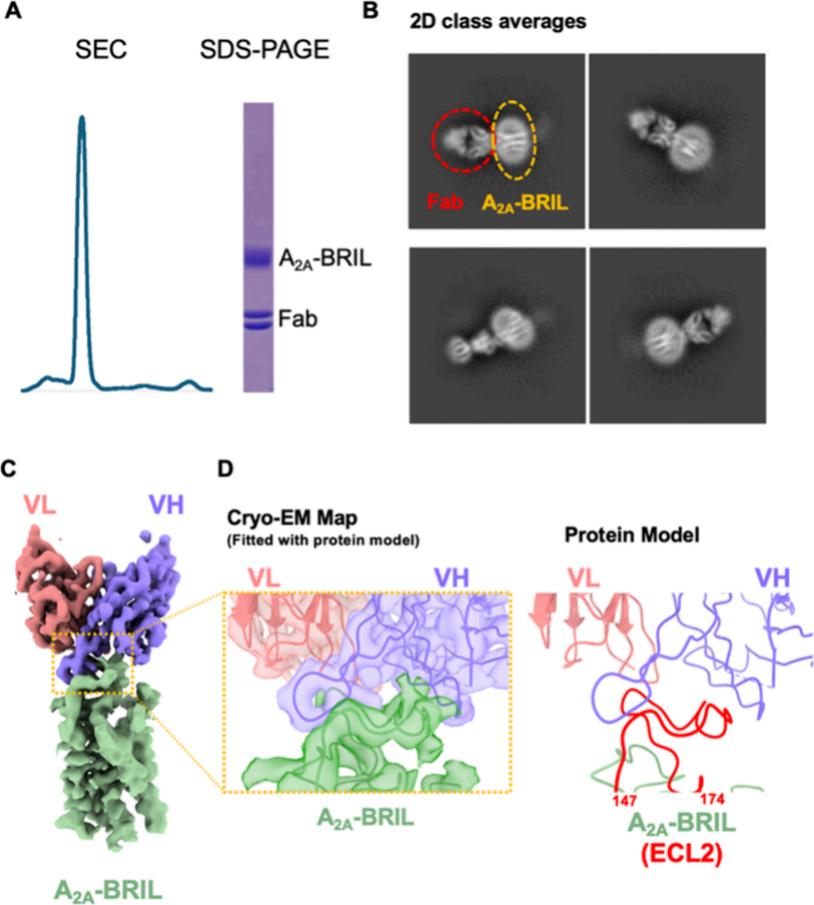
Cryo-EM of the A_2A_-BRIL-Fab complex identifies an ECL2 antibody-binding surface consistent with STLiP-MS. (A) SEC chromatogram and SDS-PAGE confirming the formation of the A_2A_-BRIL-Fab complex. (B) Representative reference-free 2D class averages showing the map attributable to Fab attached to the A_2A_-BRIL. (C) Cryo-EM map segmented into A_2A_-BRIL (green) and Fab variable domains (VL, red; VH, purple) demonstrating Fab binding on the extracellular face of the receptor. (D) Magnified view of the fitted map (left) and the corresponding atomic model (right) highlights the contacts between VH and ECL2 of A_2A_-BRIL. The interface overlaps with the protected region detected using STLiP-MS (residues 147–174), supporting ECL2 as the antibody-binding surface. Cryo-EM, cryogenic electron microscopy; STLiP-MS, Swift Trypsin-limited proteolysis–mass spectrometry; SEC, size exclusion chromatography; SDS-PAGE, sodium dodecyl sulfate-polyacrylamide gel electrophoresis; 2D, two-dimensional; ECL2, extracellular loop 2.

The complex solution was vitrified on EM grids and subjected to cryo-EM imaging, followed by a single-particle analysis. The 2D class averages showed A_2A_-BRIL bound to Fab (Figure 6B), and three-dimensional reconstruction revealed Fab binding at the extracellular side of the receptor (Figure 6C, EMDB: EMD-67107, PDB: 9XQB). Local resolution analysis indicated that the Fab variable region, particularly the VH domain, was in close contact with the ECL2 region, consistent with the STLiP-MS-detected structural change region (residues 147–176) (Figure 6C,D).

Altogether, these results demonstrate that STLiP-MS using a trypsin-immobilized spin column enables highly sensitive detection of local structural changes induced by antibody interactions at the peptide level and provides a robust approach for predicting binding sites, showing strong concordance with cryo-EM structural analysis.

## Conclusions

In this study, we established a one-step STLiP-MS protocol by implementing rapid and highly reproducible surface-limited proteolysis using a trypsin-immobilized spin column. Compared with conventional two-step digestion methods, this protocol is markedly simpler and, together with optimization of the inactivation step, provides enhanced sensitivity for detecting structural changes.

By contrasting phosphatase inhibitor-induced structural alterations with the results from the SP3 method, which involves complete denaturation and digestion, we confirmed that STLiP-MS specifically captures structure-selective proteolysis. Furthermore, the analysis of the A_2A_ receptor in complex with an IgG antibody demonstrated that STLiP-MS can sensitively detect local structural changes at the peptide level upon antibody binding. In combination with cryo-EM-based structural analysis, we verified that the STLiP-MS results accurately corresponded to the actual antibody-binding sites.

While STLiP-MS enables the sensitive detection of local, structure-dependent changes under near-physiological conditions, it has several limitations. First, trypsin’s cleavage-site bias can lead to uneven sequence coverage in Lys/Arg-poor regions and within transmembrane segments. Coverage can be complemented by orthogonal proteases (e.g., Lys-C) at the cost of added data integration complexity. Second, the spatial resolution is at the peptide-fragment level; unlike XL-MS, it does not provide absolute distance constraints, and unlike HDX-MS, it does not report exchange kinetics. Thus, interface assignment relies on “protection/sensitization fingerprints” and benefits from orthogonal confirmation by high-resolution structural methods (e.g., cryo-EM).

Collectively, these findings establish STLiP-MS as a practical platform for the comprehensive and high-precision detection of local, structure-dependent changes associated with post-translational modifications or molecular interactions under near-physiological conditions, with broad utility in structural proteomics, including biomarker discovery, drug target identification, and elucidation of structure-based mechanisms of functional regulation.

## Supplementary Material



## Data Availability

Mass spectrometry proteomics data have been deposited to the ProteomeXchange Consortium via the jPOST partner repository^26^ under the identifiers PXD09771 (ProteomeXchange) and JPST004097 (jPOST). The cryo-EM maps have been deposited in the Electron Microscopy Data Bank (EMDB) under the accession number EMD-67107. The structural coordinates have been deposited in the Protein Data Bank (PDB) under the accession number 9XQB, corresponding to the A_2A_-BRIL-Fab complex in the absence of inhibitors reported in this paper.

## Notes

A_2A_-BRIL-Fab cryo-EM processing workflow; LiP-MS peptide protection analysis; cryo-EM data summary; and protein lists showing differential trypsin cleavage before and after optimization (PDF)

## Abbreviations

MS: mass spectrometry
HDX–MS: hydrogen–deuterium exchange mass spectrometry
XL-MS: cross-linking mass spectrometry
LiP–MS: limited proteolysis–mass spectrometry
STLiP–MS: Swift trypsin-limited proteolysis–mass spectrometry
SP3: single-pot solid phase-enhanced sample preparation
LMNG: lauryl maltose neopentyl glycol
CHS: cholesteryl hemisuccinate
SDS: sodium dodecyl sulfate
TCEP: tris­(2-carboxyethyl)­phosphine
IAA: iodoacetamide
TFA: trifluoroacetic acid
ACN: acetonitrile
DMNG: dodecyl maltoside neopentyl glycol
AGC: automatic gain control
LC/MS/MS: liquid chromatography-tandem mass spectrometry
Cryo-EM: cryogenic electron microscopy
DDA: data-dependent acquisition
DIA: data-independent acquisition
PBS: phosphate-buffered saline
HEPES: 4-(2-hydroxyethyl)-1-piperazineethanesulfonic acid
SEC: size exclusion chromatography
FDR: false discovery rate
PDB: protein data bank
CTF: contrast transfer function
2D: two-dimensional
ECL2: extracellular loop 2
GO: Gene Ontology
SDS-PAGE: sodium dodecyl sulfate-polyacrylamide gel electrophoresis
