# Supplementary material for: Rapid and Label-Free Structural Proteomics Using One-Step Swift Trypsin LiP-MS
Source: ACS Omega. 2025 Dec 25;11(1):2152–62. doi: 10.1021/acsomega.5c11109 (PMC12809284; doi:10.1021/acsomega.5c11109)
Supplement: Supplementary file 1 [file ao5c11109_si_001.pdf]

Supporting Information

Rapid and Label-Free Structural Proteomics Using One-Step Swift Trypsin LiP–MS

*Yasuomi Miyashita[1,2,3], Ryo Konno[1], Satoshi Ogasawara[3,4], Yusei Okuda[1,5], Yuuki Takamuku[3], Toshio Moriya[6], Tetsuichiro Saito[2], Takeshi Murata[3,4], Osamu Ohara[1], Yusuke Kawashima[1]**

[1] Department of Applied Genomics, Kazusa DNA Research Institute, 2-6-7 Kazusa-kamatari, Kisarazu, Chiba 292-0818, Japan

[2] Department of Developmental Biology, Graduate School of Medicine, Chiba University, 1-8-1 Inohana, Chuo, Chiba 260-8670, Japan

[3] Department of Chemistry, Graduate School of Science, Chiba University, 1-33 Yayoi-cho, Inage, Chiba 263-8522, Japan

[4] Center of Quantum Life Science for Structural Therapeutics (cQUEST), Chiba University, 1-33 Yayoi-cho, Inage, Chiba 263-8522, Japan

[5] Department of Physics, School of Science, Kitasato University, 1-15-1 Kitasato, Minami-ku, Sagamihara-shi, Kanagawa 252-0373, Japan

[6] Structural Biology Research Center, Institute of Materials Structure Science, High Energy Accelerator Research Organization (KEK), 1-1 Oho, Tsukuba, Ibaraki, 305-0801, Japan

Table of Contents

Supplementary Figures

Figure S1. Cryo-EM data processing workflow for the A2A-BRIL–Fab complex
Figure S2. LiP–MS peptide profiles reveal antibody-protected regions in A2A-BRIL

Supplementary Tables

Table S1. Cryo-EM data collection, processing, and refinement summary for the A_2A-BRIL–Fab complex
Table S2. List of 286 proteins showing differential trypsin cleavage before optimization
Table S3. List of 799 proteins showing differential trypsin cleavage after optimization

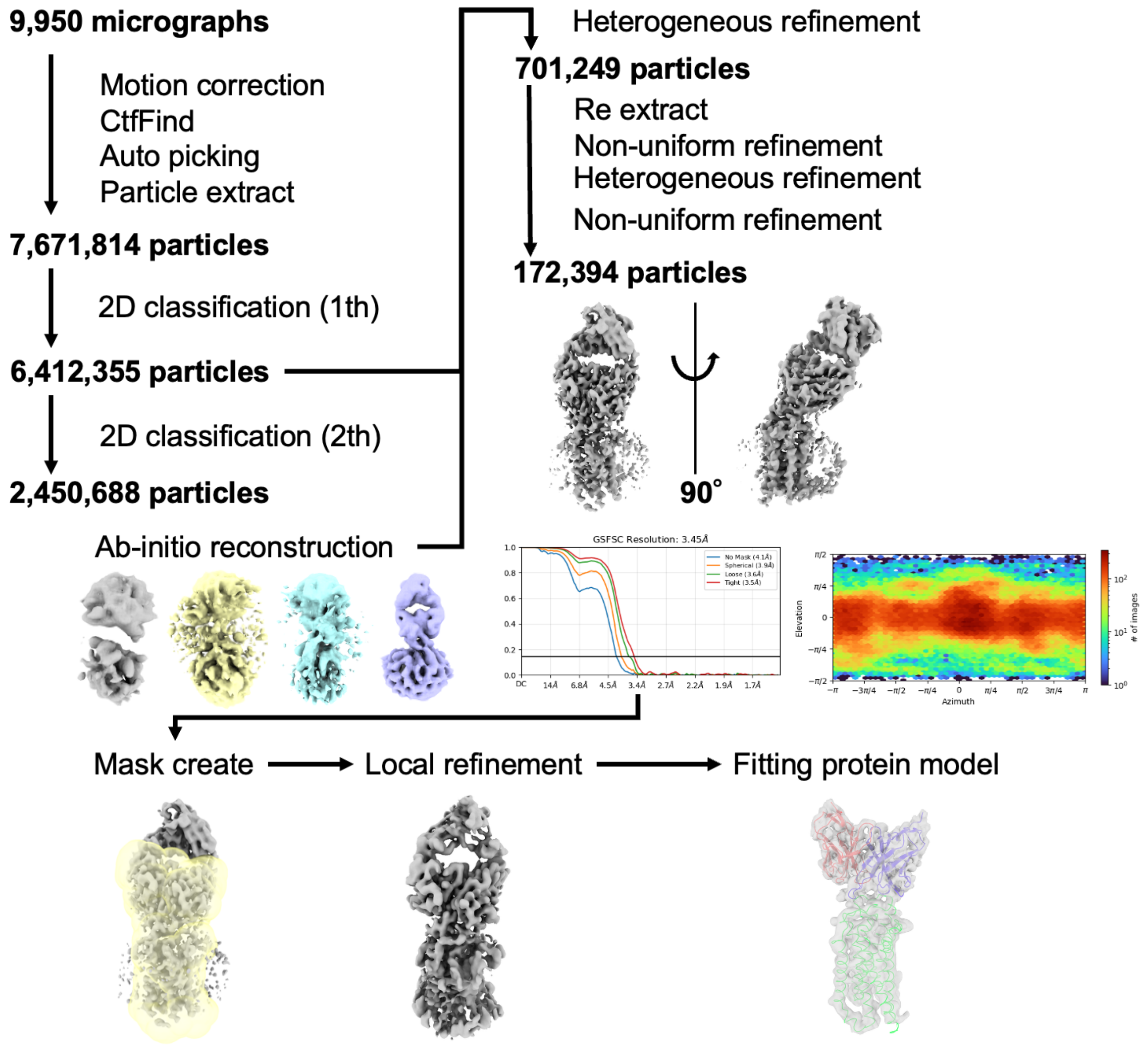

Figure S1. Cryo-EM data processing workflow for the A_{2A}-BRIL–Fab complex

Workflow of single-particle cryo-EM processing. After particle picking and 2D/3D classifications, selected particles were subjected to heterogeneous and non-uniform refinement, followed by local refinement with a focused mask to obtain the final reconstruction.

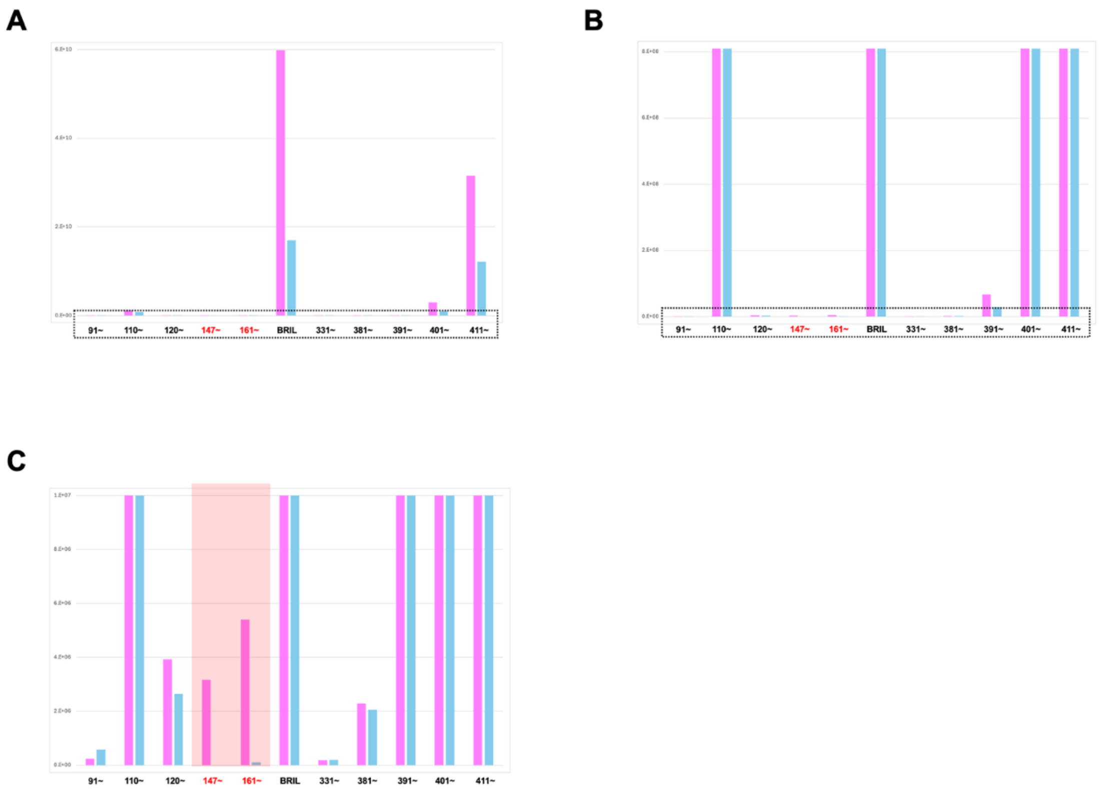

Figure S2. LiP–MS peptide profiles reveal antibody-protected regions in A_{2A}-BRIL
(A–C) Comparative LiP–MS peptide intensities for A_{2A}-BRIL alone (pink) and the A_{2A}-BRIL–IgG complex (blue). Several peptides show decreased susceptibility in the complex, most notably residues 147–160 (MLGWNNCGQPKEGK) and 161–176 (QHSQGCGEGQVACLFE), which map to the extracellular loop 2 (ECL2).

Table S1. Cryo-EM data collection, processing, and refinement summary for the A_2A-BRIL–Fab complex.

**Data collection and processing**	
**Microscope**	Titan Krios G4
**Voltage [kV]**	300
**Detector**	Falcon 4i
**Magnification**	165 k
**Pixel size [Å] (calibrated)**	0.75
**Automation software**	EPU
**Total exposure [e^-/Å^2]**	50
**Exposure rate [e^-/Å^2 frame]**	1
**Number of frames**	50
**Defocus range [μm]**	0.8 – 2.0
**Number of collected micrographs**	9,950
**No. of particles for Final map**	172,394
**Map Global Resolution [Å]**	3.41
**FSC threshold**	0.143
**Reconstruction**	
**Refinement programs**	PHENIX
**Model resolution [Å]**	3.6
**FSC threshold**	0.5

Table S2. List of 286 proteins showing differential trypsin cleavage before optimization

UniProt Accession number	Master protein name	Fold-change (phosphatase inhibitor/Contral)	Welch's *t* -test *p* -value
O75083	WD repeat-containing protein 1	144.761	4.03E-04
Q92526	T-complex protein 1 subunit zeta-2	143.845	1.85E-04
Q8TEY7	Ubiquitin carboxyl-terminal hydrolase 33	42.486	2.84E-04
Q5TF21	Protein SOGA3	42.324	8.05E-06
Q8N8L6	ADP-ribosylation factor-like protein 10	29.484	1.64E-05
P61009	Signal peptidase complex subunit 3	27.117	1.24E-04
Q9Y3A6	Transmembrane emp24 domain-containing protein 5	25.178	4.68E-03
O75884	Serine hydrolase RBBP9	25.157	1.82E-03
Q9H300	Presenilins-associated rhomboid-like protein, mitochondrial	24.788	5.22E-04
Q3KRA6	UPF0538 protein C2orf76	24.611	1.59E-03
Q9NUQ2	1-acyl-sn-glycerol-3-phosphate acyltransferase epsilon	21.832	4.16E-02
Q5VU97	VWFA and cache domain-containing protein 1	19.811	6.68E-05
P49914	5-formyltetrahydrofolate cyclo-ligase	19.556	1.60E-05
Q16539	Mitogen-activated protein kinase 14	18.598	4.25E-02
P0CG40	Transcription factor Sp9	16.746	4.04E-04
Q9UBK9	Protein UXT	15.836	2.16E-03
Q8IYN2	Transcription elongation factor A protein-like 8	15.689	5.58E-03
Q6UXN9	WD repeat-containing protein 82	15.229	2.32E-04
Q96B49	Mitochondrial import receptor subunit TOM6 homolog	14.622	3.42E-02
P18074	General transcription and DNA repair factor IIH helicase subunit XPD	14.259	3.27E-04
Q6UWP2	Dehydrogenase/reductase SDR family member 11	13.898	2.44E-05
P60604	Ubiquitin-conjugating enzyme E2 G2	13.809	3.20E-03
Q9UGN5	Poly [ADP-ribose] polymerase 2	13.518	1.73E-03
O75132	Zinc finger BED domain-containing protein 4	12.727	1.77E-03
Q8TBE9	N-acylneuraminate-9-phosphatase	12.649	3.51E-03
Q8TD08	Mitogen-activated protein kinase 15	12.345	1.02E-02
P19971	Thymidine phosphorylase	11.655	2.66E-05
Q9Y2D8	Afadin- and alpha-actinin-binding protein	11.208	5.57E-03
Q15269	Periodic tryptophan protein 2 homolog	10.803	1.62E-02
Q96FJ0	AMSH-like protease	10.720	3.45E-03
P14927	Cytochrome b-c1 complex subunit 7	10.349	5.69E-03
O14508	Suppressor of cytokine signaling 2	9.793	9.37E-06
P26022	Pentraxin-related protein PTX3	9.601	4.87E-03
Q8WV41	Sorting nexin-33	9.600	3.27E-02
P60953	Cell division control protein 42 homolog	9.104	3.85E-02
Q9UL49	Transcription factor-like 5 protein	8.816	5.23E-03
Q9HCC6	Transcription factor HES-4	8.765	2.99E-02
Q13887	Krueppel-like factor 5	8.600	7.13E-04
Q9NW97	Transmembrane protein 51	8.492	4.08E-02
O95755	Ras-related protein Rab-36	8.463	1.54E-02
Q9BV35	Calcium-binding mitochondrial carrier protein SCaMC-3	8.457	2.93E-02
Q2TAA2	Isoamyl acetate-hydrolyzing esterase 1 homolog	8.129	4.45E-04
Q5SWX8	Protein odr-4 homolog	8.126	2.41E-03
Q9UNL4	Inhibitor of growth protein 4	8.018	1.88E-03
Q9BUM1	Glucose-6-phosphatase 3	7.915	5.71E-07
Q9Y315	Deoxyribose-phosphate aldolase	7.877	5.67E-04
O14656	Torsin-1A	7.861	1.10E-02
Q16206	Ecto-NOX disulfide-thiol exchanger 2	7.853	3.75E-03
P78417	Glutathione S-transferase omega-1	7.774	3.70E-02
Q8IWB1	Inositol 1,4,5-trisphosphate receptor-interacting protein	7.502	2.16E-02
Q8IW41	MAP kinase-activated protein kinase 5	7.276	1.92E-02
Q96PE7	Methylmalonyl-CoA epimerase, mitochondrial	7.170	3.60E-02
P00441	Superoxide dismutase [Cu-Zn]	7.161	2.12E-02
P54278	Mismatch repair endonuclease PMS2	6.886	1.08E-02
Q1ZZU3	DNA repair protein SWI5 homolog	6.843	4.84E-02
P52630	Signal transducer and activator of transcription 2	6.720	1.22E-02
Q9NXB0	Meckel syndrome type 1 protein	6.668	4.81E-02
O15270	Serine palmitoyltransferase 2	6.572	2.54E-03
Q9BZG8	2-(3-amino-3-carboxypropyl)histidine synthase subunit 1	6.556	6.87E-05
P03886	NADH-ubiquinone oxidoreductase chain 1	6.416	1.20E-03
Q9Y3V2	RWD domain-containing protein 3	6.403	9.87E-04
Q9NX38	Protein Abitram	6.349	2.47E-02
Q71RG4	Transmembrane and ubiquitin-like domain-containing protein 2	6.095	3.38E-02
Q9Y5L0	Transportin-3	6.078	2.13E-02
Q96JN8	Neuralized-like protein 4	5.947	4.49E-03
A5PL33	Protein KRBA1	5.836	1.46E-02
P28070	Proteasome subunit beta type-4	5.736	3.24E-02
Q969E8	Pre-rRNA-processing protein TSR2 homolog	5.641	8.36E-03
Q9ULS5	Transmembrane and coiled-coil domain protein 3	5.625	1.02E-03
Q14469	Transcription factor HES-1	5.592	2.43E-02
Q9ULC4	Malignant T-cell-amplified sequence 1	5.577	1.09E-02
Q8IZV5	Retinol dehydrogenase 10	5.530	3.51E-02
Q99497	Parkinson disease protein 7	5.465	3.92E-08
Q96A22	Uncharacterized protein C11orf52	5.464	2.87E-02
Q8WTR2	Dual specificity protein phosphatase 19	5.417	6.26E-03
Q8N961	Ankyrin repeat and BTB/POZ domain-containing protein 2	5.385	2.59E-03
Q9H1E3	Nuclear ubiquitous casein and cyclin-dependent kinase substrate 1	5.317	1.09E-05
P62834	Ras-related protein Rap-1A	5.306	2.75E-04
Q8IX21	SMC5-SMC6 complex localization factor protein 2	5.291	3.68E-02
Q5JU69	Torsin-2A	5.161	1.08E-02
P83916	Chromobox protein homolog 1	5.132	2.90E-02
P43034	Platelet-activating factor acetylhydrolase IB subunit alpha	5.066	2.02E-02
O95865	N(G),N(G)-dimethylarginine dimethylaminohydrolase 2	5.057	1.45E-02
Q14728	Major facilitator superfamily domain-containing protein 10	5.055	2.42E-03

Q99865;Q9BPZ2	Spindlin-2A	5.048	1.41E-02
Q53H82	Endoribonuclease LACTB2	4.974	3.74E-03
P42575	Caspase-2	4.831	5.95E-03
Q9NQ88	Fructose-2,6-bisphosphatase TIGAR	4.799	1.17E-09
Q9P0U1	Mitochondrial import receptor subunit TOM7 homolog	4.779	4.28E-05
Q9NU53	Glycoprotein integral membrane protein 1	4.747	2.99E-02
Q5EE01	Centromere protein W	4.645	8.62E-03
Q9BQ67	Glutamate-rich WD repeat-containing protein 1	4.579	2.17E-02
P18669	Phosphoglycerate mutase 1	4.559	2.53E-06
Q8N9A8	Nuclear envelope phosphatase-regulatory subunit 1	4.476	5.23E-03
Q9Y3E7	Charged multivesicular body protein 3	4.471	9.79E-03
P15260	Interferon gamma receptor 1	4.469	9.72E-03
Q9P2K1	Coiled-coil and C2 domain-containing protein 2A	4.443	3.66E-02
Q9NVM4	Protein arginine N-methyltransferase 7	4.420	2.16E-02
P00374	Dihydrofolate reductase	4.419	5.63E-06
O95861	3'(2'),5'-bisphosphate nucleotidase 1	4.346	2.76E-05
Q92508	Piezo-type mechanosensitive ion channel component 1	4.244	1.45E-02
Q9Y2G8	DnaJ homolog subfamily C member 16	4.190	3.22E-02
O94986	Centrosomal protein of 152 kDa	4.184	1.42E-02
P11117	Lysosomal acid phosphatase	4.181	3.59E-03
A8MT69	Centromere protein X	4.146	4.17E-04
Q9UKT7	F-box/LRR-repeat protein 3	4.138	4.00E-03
Q58FG0	Putative heat shock protein HSP 90-alpha A5	4.102	3.36E-02
P05023	Sodium/potassium-transporting ATPase subunit alpha-1	4.058	5.79E-09
Q9P2G1	Ankyrin repeat and IBR domain-containing protein 1	4.021	1.44E-02
Q9H074	Polyadenylate-binding protein-interacting protein 1	4.004	9.48E-05
Q9H813	Proton-activated chloride channel	3.984	1.05E-02
P81605	Dermcidin	3.980	4.47E-02
Q7Z736	Pleckstrin homology domain-containing family H member 3	3.973	3.08E-02
P04406	Glyceraldehyde-3-phosphate dehydrogenase	3.971	4.55E-11
Q15772	Striated muscle preferentially expressed protein kinase	3.839	2.49E-02
Q9NTJ5	Phosphatidylinositide phosphatase SAC1	3.804	1.39E-03
Q06520	Bile salt sulfotransferase	3.793	1.80E-02
Q9H0A8	COMM domain-containing protein 4	3.759	1.07E-02
Q9NRP0	Oligosaccharyltransferase complex subunit OSTC	3.755	1.16E-02
Q9NVP2	Histone chaperone ASF1B	3.750	2.92E-03
Q9NY33	Dipeptidyl peptidase 3	3.734	2.22E-02
O76064	E3 ubiquitin-protein ligase RNF8	3.684	4.26E-02
O95336	6-phosphogluconolactonase	3.633	6.49E-06
P11766	Alcohol dehydrogenase class-3	3.631	9.39E-05
Q14435	Polypeptide N-acetylgalactosaminyltransferase 3	3.630	1.97E-02
P24588	A-kinase anchor protein 5	3.558	4.87E-02
P09972	Fructose-bisphosphate aldolase C	3.525	1.82E-02
Q9Y394	Dehydrogenase/reductase SDR family member 7	3.516	1.58E-05
Q9NRG1	Phosphoribosyltransferase domain-containing protein 1	3.514	5.31E-03
Q7Z6J8	E3 ubiquitin-protein ligase E3D	3.504	3.64E-02
Q9BY49	Peroxisomal trans-2-enoyl-CoA reductase	3.502	1.65E-06
Q9BSH5	Haloacid dehalogenase-like hydrolase domain-containing protein 3	3.499	7.46E-07
Q9NX04	Uncharacterized protein C1orf109	3.498	4.76E-02
Q7L8J4	SH3 domain-binding protein 5-like	3.419	1.42E-02
Q9BQG2	Peroxisomal NADH pyrophosphatase NUDT12	3.364	1.70E-03
P17509	Homeobox protein Hox-B6	3.332	5.23E-06
Q9GZP9	Derlin-2	3.310	1.70E-02
O75071	EF-hand calcium-binding domain-containing protein 14	3.297	6.46E-03
O15264	Mitogen-activated protein kinase 13	3.296	1.22E-02
P51452	Dual specificity protein phosphatase 3	3.293	3.33E-07
P61916	NPC intracellular cholesterol transporter 2	3.237	5.41E-05
P48449	Lanosterol synthase	3.216	1.41E-02
P62837	Ubiquitin-conjugating enzyme E2 D2	3.143	4.63E-02
Q96GJ1	tRNA (uracil(54)-C(5))-methyltransferase homolog	3.127	6.84E-03
Q86W42	THO complex subunit 6 homolog	3.066	1.29E-02
Q8N0U8	Vitamin K epoxide reductase complex subunit 1-like protein 1	3.052	2.52E-02
Q6NTF9	Rhomboid domain-containing protein 2	3.046	2.22E-02
Q9NZD8	Maspardin	3.003	1.05E-02
P35237	Serpin B6	2.988	3.56E-02
Q9BY41	Histone deacetylase 8	2.936	1.43E-02
Q9NZB8	Molybdenum cofactor biosynthesis protein 1	2.935	4.16E-02
Q6P1M9	Armadillo repeat-containing X-linked protein 5	2.930	4.55E-02
Q8WY54	Protein phosphatase 1E	2.922	3.34E-03
Q8WYK2	Jun dimerization protein 2	2.867	2.77E-02
A6NDG6	Glycerol-3-phosphate phosphatase	2.838	2.54E-02
P29218	Inositol monophosphatase 1	2.829	4.41E-06
Q9Y2V2	Calcium-regulated heat-stable protein 1	2.812	3.71E-07
P08651	Nuclear factor 1 C-type	2.794	1.60E-02
Q5BKY9;Q8N9E0	Protein FAM133B	2.787	2.38E-05
Q15819	Ubiquitin-conjugating enzyme E2 variant 2	2.774	8.49E-06
Q9NXK8	F-box/LRR-repeat protein 12	2.754	1.57E-02
Q9H0F7	ADP-ribosylation factor-like protein 6	2.750	4.87E-02
Q9NUJ1	Mycophenolic acid acyl-glucuronide esterase, mitochondrial	2.749	1.98E-02
Q96RQ3	Methylcrotonoyl-CoA carboxylase subunit alpha, mitochondrial	2.729	9.60E-03
Q96BW1	Uracil phosphoribosyltransferase homolog	2.723	1.63E-02
Q9HAD4	WD repeat-containing protein 41	2.716	1.34E-04
P07738	Bisphosphoglycerate mutase	2.709	4.04E-02
Q9BV20	Methylthioribose-1-phosphate isomerase	2.691	1.44E-06
Q8NFI3	Cytosolic endo-beta-N-acetylglucosaminidase	2.688	5.16E-08
Q13740	CD166 antigen	2.681	5.21E-03
O75340	Programmed cell death protein 6	2.675	3.02E-05
P03928	ATP synthase protein 8	2.673	3.52E-06

O43734	Adapter protein CIKS	2.659	8.83E-04
Q96JI7	Spatacsin	2.658	3.86E-03
Q562R1	Beta-actin-like protein 2	2.653	1.13E-05
O15514	DNA-directed RNA polymerase II subunit RPB4	2.652	3.91E-06
Q96FW1	Ubiquitin thioesterase OTUB1	2.644	3.20E-05
P78330	Phosphoserine phosphatase	2.631	3.02E-08
O96011	Peroxisomal membrane protein 11B	2.596	1.91E-06
Q9H6R3	Acyl-CoA synthetase short-chain family member 3, mitochondrial	2.586	1.32E-03
Q4ZIN3	Membralin	2.577	8.51E-03
Q13547	Histone deacetylase 1	2.557	8.76E-06
Q96ND0	Protein FAM210A	2.555	1.87E-03
Q7Z4H3	5'-deoxynucleotidase HDDC2	2.549	5.57E-05
Q8N9F7	Lysophospholipase D GDPD1	2.535	1.03E-02
P60709	Actin, cytoplasmic 1	2.530	1.99E-07
Q96A46	Mitoferrin-2	2.529	4.44E-02
Q96JM2	Zinc finger protein 462	2.527	6.73E-03
Q9UHQ4	B-cell receptor-associated protein 29	2.526	4.87E-02
P07099	Epoxide hydrolase 1	2.520	3.75E-02
Q13838	Spliceosome RNA helicase DDX39B	2.508	1.73E-09
P19838	Nuclear factor NF-kappa-B p105 subunit	2.501	3.81E-02
Q9NTK5	Obg-like ATPase 1	2.485	1.41E-06
P21399	Cytoplasmic aconitate hydratase	2.483	2.22E-04
O15525	Transcription factor MafG	2.482	3.50E-04
P54819	Adenylate kinase 2, mitochondrial	2.480	8.29E-10
P31949	Protein S100-A11	2.477	2.34E-05
Q9UGL1	Lysine-specific demethylase 5B	2.466	3.99E-02
Q9NPJ3	Acyl-coenzyme A thioesterase 13	2.462	1.77E-03
Q13404	Ubiquitin-conjugating enzyme E2 variant 1	2.462	2.46E-05
Q9H902	Receptor expression-enhancing protein 1	2.458	1.66E-03
O75323	Protein NipSnap homolog 2	2.455	2.91E-04
P05771	Protein kinase C beta type	2.445	2.88E-02
Q02539	Histone H1.1	2.441	1.93E-06
Q9HC38	Glyoxalase domain-containing protein 4	2.440	1.03E-03
Q15404	Ras suppressor protein 1	2.425	1.40E-03
Q8NFZ0	F-box DNA helicase 1	2.411	8.11E-03
Q5JRX3	Presequence protease, mitochondrial	2.395	1.72E-06
Q8IVL6	Prolyl 3-hydroxylase 3	2.390	1.63E-02
P98174	FYVE, RhoGEF and PH domain-containing protein 1	2.378	4.12E-04
Q93045	Stathmin-2	2.360	1.04E-05
Q9Y3C8	Ubiquitin-fold modifier-conjugating enzyme 1	2.358	8.59E-06
O43513	Mediator of RNA polymerase II transcription subunit 7	2.358	4.12E-03
Q9NRN7	L-aminoadipate-semialdehyde dehydrogenase-phosphopantetheinyl transferase	2.349	4.09E-06
Q00169	Phosphatidylinositol transfer protein alpha isoform	2.345	2.58E-02
Q99653	Calcineurin B homologous protein 1	2.334	2.29E-03
P04062	Lysosomal acid glucosylceramidase	2.330	1.53E-02
Q9P287	BRCA2 and CDKN1A-interacting protein	2.325	1.65E-02
Q9NX46	ADP-ribose glycohydrolase ARH3	2.319	7.09E-06
P15121	Aldo-keto reductase family 1 member B1	2.304	1.23E-04
Q16222	UDP-N-acetylhexosamine pyrophosphorylase	2.299	4.04E-05
Q13526	Peptidyl-prolyl cis-trans isomerase NIMA-interacting 1	2.298	5.32E-08
Q8WZ82	Esterase OVCA2	2.293	1.18E-05
Q8N4P3	Guanosine-3',5'-bis(diphosphate) 3'-pyrophosphohydrolase MESH1	2.289	2.50E-04
Q13131	5'-AMP-activated protein kinase catalytic subunit alpha-1	2.256	1.25E-05
Q86SQ9	Dehydrodolichyl diphosphate synthase complex subunit DHDDS	2.253	4.01E-02
Q15005	Signal peptidase complex subunit 2	2.247	1.23E-06
O14924	Regulator of G-protein signaling 12	2.246	1.19E-02
Q9UEW8	STE20/SPS1-related proline-alanine-rich protein kinase	2.245	9.76E-04
P24666	Low molecular weight phosphotyrosine protein phosphatase	2.235	6.23E-07
Q6NSJ2	Pleckstrin homology-like domain family B member 3	2.227	3.34E-02
Q99536	Synaptic vesicle membrane protein VAT-1 homolog	2.226	1.07E-06
P49761	Dual specificity protein kinase CLK3	2.225	2.12E-05
P18085	ADP-ribosylation factor 4	2.222	1.74E-03
P51589	Cytochrome P450 2J2	2.221	5.68E-03
P06132	Uroporphyrinogen decarboxylase	2.219	2.86E-06
Q96C86	m7GpppX diphosphatase	2.218	2.59E-03
P07311	Acylphosphatase-1	2.216	3.22E-05
Q8TBZ3	WD repeat-containing protein 20	2.212	1.05E-03
Q15043	Metal cation symporter ZIP14	2.209	1.75E-02
Q96HE7	ERO1-like protein alpha	2.207	2.20E-03
P09455	Retinol-binding protein 1	2.184	2.58E-02
P49770	Translation initiation factor eIF-2B subunit beta	2.179	2.86E-07
Q6UW68	Transmembrane protein 205	2.174	1.18E-06
Q6GMV3	Putative peptidyl-tRNA hydrolase PTRHD1	2.174	1.74E-02
Q96EH3	Mitochondrial assembly of ribosomal large subunit protein 1	2.172	4.76E-03
O94972	E3 ubiquitin-protein ligase TRIM37	2.153	1.16E-04
P84077	ADP-ribosylation factor 1	2.153	8.20E-06
Q6NYC1	Bifunctional arginine demethylase and lysyl-hydroxylase JMJD6	2.151	1.30E-04
O15173	Membrane-associated progesterone receptor component 2	2.146	1.48E-07
Q8TEP8	Centrosomal protein of 192 kDa	2.119	2.15E-05
Q969G6	Riboflavin kinase	2.114	5.55E-07
P09211	Glutathione S-transferase P	2.109	1.95E-08
Q9HAB8	Phosphopantothenate--cysteine ligase	2.108	7.27E-05
Q9NRX5	Serine incorporator 1	2.100	9.53E-06
Q9Y5L4	Mitochondrial import inner membrane translocase subunit Tim13	2.093	4.64E-06
O75608	Acyl-protein thioesterase 1	2.089	1.07E-03
Q96IG2	F-box/LRR-repeat protein 20	2.089	1.46E-03
P00505	Aspartate aminotransferase, mitochondrial	2.086	5.17E-07
Q9UNF0	Protein kinase C and casein kinase substrate in neurons protein 2	2.084	9.84E-05

O60488	Long-chain-fatty-acid--CoA ligase 4	2.080	1.22E-04
P28482	Mitogen-activated protein kinase 1	2.072	2.45E-06
Q9BXW7	Haloacid dehalogenase-like hydrolase domain-containing 5	2.067	6.03E-05
Q06323	Proteasome activator complex subunit 1	2.063	6.01E-06
Q9NP77	RNA polymerase II subunit A C-terminal domain phosphatase SSU72	2.059	5.45E-03
Q8N954	G patch domain-containing protein 11	2.056	1.57E-06
P62888	60S ribosomal protein L30	2.054	3.61E-05
P61204	ADP-ribosylation factor 3	2.053	5.34E-04
P54709	Sodium/potassium-transporting ATPase subunit beta-3	2.049	1.17E-03
Q9NZL4	Hsp70-binding protein 1	2.045	1.26E-05
P41162	ETS translocation variant 3	2.040	1.39E-06
Q86VD7	Mitochondrial coenzyme A transporter SLC25A42	2.036	1.22E-04
Q9BYM8	RanBP-type and C3HC4-type zinc finger-containing protein 1	2.035	2.04E-02
P08758	Annexin A5	2.035	7.54E-10
A0A0U1RRE5	Negative regulator of P-body association	2.031	1.56E-05
P16615	Sarcoplasmic/endoplasmic reticulum calcium ATPase 2	2.030	1.12E-06
Q8NB49	Phospholipid-transporting ATPase IG	2.030	3.26E-02
P57088	Transmembrane protein 33	2.030	4.64E-07
A0A0B4J2F0	Protein PIGBOS1	2.021	2.65E-02
P61024	Cyclin-dependent kinases regulatory subunit 1	2.019	9.82E-03
Q9Y265	RuvB-like 1	2.018	4.71E-07
Q5BJH2	Transmembrane protein 128	2.012	1.88E-02
Q9H8K7	ATPase PAAT	2.010	9.64E-04
O94813	Slit homolog 2 protein	2.003	7.19E-07
P63241	Eukaryotic translation initiation factor 5A-1	2.002	1.27E-10

Table S3. List of 799 proteins showing differential trypsin cleavage after optimization

UniProt Accession number	Master protein name	Fold-change (phosphatase inhibitor/Contral)	Welch's *t*-test *p*-value
Q8NB25	Protein FAM184A	111.328	1.2.E-03
P52951	Homeobox protein GBX-2	109.530	4.3.E-04
P31937	3-hydroxyisobutyrate dehydrogenase, mitochondrial	38.565	2.6.E-04
Q9H4W6	Transcription factor COE3	34.828	6.1.E-04
P25208	Nuclear transcription factor Y subunit beta	34.316	4.9.E-04
P35754	Glutaredoxin-1	27.083	5.1.E-03
Q96EP1	E3 ubiquitin-protein ligase CHFR	24.968	3.0.E-04
A3KMH1	von Willebrand factor A domain-containing protein 8	24.735	9.9.E-06
A1L170	Uncharacterized protein C1orf226	24.536	2.8.E-03
Q8TD55	Pleckstrin homology domain-containing family O member 2	23.305	9.9.E-04
Q63HN8	E3 ubiquitin-protein ligase RNF213	20.663	2.3.E-02
Q8WXX5	DnaJ homolog subfamily C member 9	20.114	9.0.E-04
O95772	STARD3 N-terminal-like protein	19.375	3.2.E-02
Q8N9N7	Leucine-rich repeat-containing protein 57	18.862	3.2.E-04
Q9UNI6	Dual specificity protein phosphatase 12	18.712	3.6.E-03
Q13426	DNA repair protein XRCC4	18.266	4.2.E-02
P42773	Cyclin-dependent kinase 4 inhibitor C	17.715	8.2.E-03
O43291	Kunitz-type protease inhibitor 2	17.139	3.6.E-02
Q9C0B6	BMP/retinoic acid-inducible neural-specific protein 2	16.696	9.4.E-03
P61204	ADP-ribosylation factor 3	15.509	4.5.E-02
P62256	Ubiquitin-conjugating enzyme E2 H	14.962	2.3.E-05
Q8TDC3	Serine/threonine-protein kinase BRSK1	14.931	1.5.E-03
P33527	Multidrug resistance-associated protein 1	14.464	1.7.E-03
Q92600	CCR4-NOT transcription complex subunit 9	14.319	1.2.E-02
Q9Y5V0	Zinc finger protein 706	13.727	8.3.E-03
Q9UGR2	Zinc finger CCCH domain-containing protein 7B	13.380	2.4.E-02
Q13535	Serine/threonine-protein kinase ATR	13.327	7.7.E-05
Q13404	Ubiquitin-conjugating enzyme E2 variant 1	13.230	1.4.E-04
Q9HDC5	Junctophilin-1	13.179	9.8.E-04
Q9H173	Nucleotide exchange factor SIL1	12.832	1.7.E-03
Q9NZN3	EH domain-containing protein 3	12.428	2.7.E-04
O60237	Protein phosphatase 1 regulatory subunit 12B	11.961	1.4.E-03
P30086	Phosphatidylethanolamine-binding protein 1	11.772	4.8.E-04
Q8NHP6	Motile sperm domain-containing protein 2	11.653	2.5.E-06
Q8N9M5	Transmembrane protein 102	11.499	1.0.E-03
O75340	Programmed cell death protein 6	11.221	1.3.E-02
P36871	Phosphoglucomutase-1	11.050	3.5.E-02
P22413	Ectonucleotide pyrophosphatase/phosphodiesterase family member 1	10.862	1.9.E-02
Q9C040	Tripartite motif-containing protein 2	10.644	9.5.E-04
Q9H0R4	Haloacid dehalogenase-like hydrolase domain-containing protein 2	10.610	5.8.E-05
Q9P0M6	Core histone macro-H2A.2	10.376	3.3.E-05
O94953	Lysine-specific demethylase 4B	10.215	1.0.E-02
Q659C4	La-related protein 1B	10.120	7.8.E-05
O95503	Chromobox protein homolog 6	10.094	1.0.E-03
P13647	Keratin, type II cytoskeletal 5	10.094	3.7.E-02
Q4KMQ2	Anoctamin-6	9.964	2.0.E-02
Q9NWL6	Asparagine synthetase domain-containing protein 1	9.919	5.9.E-06
Q7Z2Z1	Treslin	9.907	5.2.E-03
Q13114	TNF receptor-associated factor 3	9.678	1.0.E-02
P28799	Progranulin	9.559	1.1.E-03
Q6UX53	Thiol S-methyltransferase TMT1B	9.371	1.6.E-03
Q9P1U1	Actin-related protein 3B	9.298	2.9.E-02
Q9NZC7	WW domain-containing oxidoreductase	9.283	2.7.E-02
Q5SW96	Low density lipoprotein receptor adapter protein 1	9.044	7.2.E-04
Q8IV08	5'-3' exonuclease PLD3	9.023	5.5.E-04
Q5VST9	Obscurin	8.822	7.1.E-04
O15084	Serine/threonine-protein phosphatase 6 regulatory ankyrin repeat subunit A	8.779	2.3.E-06
P51452	Dual specificity protein phosphatase 3	8.584	1.9.E-07
A4D1U4	DENN domain-containing protein 11	8.429	1.2.E-02
O60641	Clathrin coat assembly protein AP180	8.427	6.1.E-05
Q14571	Inositol 1,4,5-trisphosphate-gated calcium channel ITPR2	8.249	1.5.E-02
A2RU67	Protein FAM234B	8.249	6.9.E-03
Q7Z412	Peroxisome assembly protein 26	8.115	4.3.E-03
P35226	Polycomb complex protein BMI-1	8.062	1.8.E-03
O95865	Putative hydrolase DDAH2	7.988	7.2.E-03
A8MT33	Synaptonemal complex central element protein 1-like	7.929	7.9.E-03
P16298	Serine/threonine-protein phosphatase 2B catalytic subunit beta isoform	7.789	3.1.E-02
Q99607	ETS-related transcription factor Elf-4	7.705	1.5.E-07
O43184	Disintegrin and metalloproteinase domain-containing protein 12	7.517	1.1.E-02
P67812	Signal peptidase complex catalytic subunit SEC11A	7.513	9.1.E-05
P60983	Glia maturation factor beta	7.408	1.1.E-04
Q6GMV3	Putative peptidyl-tRNA hydrolase PTRHD1	7.302	1.5.E-02
Q7L592	Protein arginine methyltransferase NDUFAF7, mitochondrial	7.299	4.0.E-03
Q15910	Histone-lysine N-methyltransferase EZH2	7.269	3.1.E-04
Q96IZ5	RNA-binding protein 41	7.258	9.0.E-05
Q9H9Y4	GPN-loop GTPase 2	7.220	3.2.E-02
Q9H9H4	Vacuolar protein sorting-associated protein 37B	7.189	5.1.E-05
Q9UDX5	Mitochondrial fission process protein 1	7.051	2.5.E-02
O43592	Exportin-T	7.010	2.7.E-03
O14668	Transmembrane gamma-carboxyglutamic acid protein 1	6.979	8.4.E-04
Q99583	Max-binding protein MNT	6.910	5.8.E-04
Q9NUM4	Transmembrane protein 106B	6.887	2.0.E-02
O95881	Thioredoxin domain-containing protein 12	6.848	3.6.E-02
Q9NUB1	Acetyl-coenzyme A synthetase 2-like, mitochondrial	6.830	1.2.E-02

P17676	CCAAT/enhancer-binding protein beta	6.702	1.1.E-02
P10599	Thioredoxin	6.662	3.7.E-03
O75387	Large neutral amino acids transporter small subunit 3	6.584	1.7.E-03
Q7Z7H5	Transmembrane emp24 domain-containing protein 4	6.578	4.1.E-02
Q5VYS8	Terminal uridylyltransferase 7	6.550	3.3.E-03
Q8N5Z5	BTB/POZ domain-containing protein KCTD17	6.516	5.6.E-03
Q9H469	F-box/LRR-repeat protein 15	6.515	6.1.E-04
P17931	Galectin-3	6.421	3.3.E-02
Q9Y4C0	Neurexin-3	6.352	6.8.E-04
Q03393	6-pyruvoyl tetrahydrobiopterin synthase	6.214	3.1.E-02
Q16537	Serine/threonine-protein phosphatase 2A 56 kDa regulatory subunit epsilon isoform	6.203	1.7.E-02
Q92930	Ras-related protein Rab-8B	6.079	1.2.E-02
Q9H8H3	Thiol S-methyltransferase TMT1A	6.052	9.7.E-04
Q9NXU5	ADP-ribosylation factor-like protein 15	5.977	4.9.E-03
Q9BTL3	RNA guanine-N7 methyltransferase activating subunit	5.908	1.4.E-07
O15318	DNA-directed RNA polymerase III subunit RPC7	5.904	7.5.E-06
Q8N128	Protein FAM177A1	5.846	3.3.E-03
P82664	Small ribosomal subunit protein uS10m	5.784	2.3.E-05
Q86UN3	Reticulon-4 receptor-like 2	5.783	1.1.E-02
P38435	Vitamin K-dependent gamma-carboxylase	5.736	1.3.E-02
Q6ZN55	Zinc finger protein 574	5.625	4.5.E-02
Q8N4J0	Carnosine N-methyltransferase	5.623	6.2.E-07
P36383	Gap junction gamma-1 protein	5.617	2.5.E-02
Q9Y2Q5	Ragulator complex protein LAMTOR2	5.608	5.9.E-04
Q8N1S5	Zinc transporter ZIP11	5.599	3.0.E-02
Q9H079	KATNB1-like protein 1	5.599	1.3.E-02
P11166	Solute carrier family 2, facilitated glucose transporter member 1	5.585	7.1.E-05
Q9P0N9	TBC1 domain family member 7	5.442	1.0.E-02
Q9Y651	Transcription factor SOX-21	5.329	8.9.E-05
Q99624	Sodium-coupled neutral amino acid transporter 3	5.316	1.7.E-02
Q96AJ1	Clusterin-associated protein 1	5.260	1.1.E-02
Q9BWW4	Single-stranded DNA-binding protein 3	5.256	3.9.E-03
Q8IWT3	Cullin-9	5.253	4.6.E-02
Q9BVW5	TIMELESS-interacting protein	5.207	1.7.E-02
Q9Y3C5	RING finger protein 11	5.199	1.4.E-03
P08134	Rho-related GTP-binding protein RhoC	5.179	8.0.E-03
Q8NI17	Interleukin-31 receptor subunit alpha	5.175	3.4.E-05
Q9BV19	Uncharacterized protein C1orf50	5.155	3.2.E-03
P78381	UDP-galactose translocator	5.079	4.5.E-02
Q99719	Septin-5	5.078	1.4.E-06
Q86SZ2	Trafficking protein particle complex subunit 6B	5.000	3.6.E-03
O60704	Protein-tyrosine sulfotransferase 2	4.977	1.1.E-02
Q9P0I2	ER membrane protein complex subunit 3	4.972	3.3.E-03
Q9NYP7	Very long chain fatty acid elongase 5	4.931	1.9.E-03
Q99471	Prefoldin subunit 5	4.929	1.5.E-02
O15239	NADH dehydrogenase [ubiquinone] 1 alpha subcomplex subunit 1	4.898	1.1.E-06
Q9HCM1	Retroelement silencing factor 1	4.888	6.7.E-03
Q9GZT6	Coiled-coil domain-containing protein 90B, mitochondrial	4.888	4.1.E-02
Q969H6	Ribonuclease P/MRP protein subunit POP5	4.880	7.3.E-03
Q9HCM4	Band 4.1-like protein 5	4.869	1.8.E-02
Q99653	Calcineurin B homologous protein 1	4.866	3.2.E-02
Q9UIF7	Adenine DNA glycosylase	4.834	5.5.E-04
Q70IA6	MOB kinase activator 2	4.771	2.1.E-02
Q9NW75	G patch domain-containing protein 2	4.763	5.4.E-04
Q9C093	Sperm flagellar protein 2	4.755	1.7.E-04
P18077	Large ribosomal subunit protein eL33	4.735	4.8.E-02
Q8WVL7	Ankyrin repeat domain-containing protein 49	4.725	1.4.E-02
P53609	Geranylgeranyl transferase type-1 subunit beta	4.722	1.3.E-02
P52306	Rap1 GTPase-GDP dissociation stimulator 1	4.710	4.1.E-05
P61925	cAMP-dependent protein kinase inhibitor alpha	4.662	1.8.E-02
Q96IX5	ATP synthase membrane subunit K, mitochondrial	4.646	2.1.E-02
Q6RW13	Type-1 angiotensin II receptor-associated protein	4.602	3.5.E-06
P14927	Cytochrome b-c1 complex subunit 7	4.590	2.1.E-02
Q9NR82	Potassium voltage-gated channel subfamily KQT member 5	4.589	1.9.E-02
Q9UBI1	COMM domain-containing protein 3	4.588	1.3.E-02
P37268	Squalene synthase	4.525	1.3.E-02
Q8WTV0	Scavenger receptor class B member 1	4.502	3.7.E-02
Q9GZX9	Twisted gastrulation protein homolog 1	4.500	9.6.E-03
Q8NC54	Keratinocyte-associated transmembrane protein 2	4.495	1.8.E-03
Q9NWA0	Mediator of RNA polymerase II transcription subunit 9	4.457	5.7.E-05
Q9Y5L4	Mitochondrial import inner membrane translocase subunit Tim13	4.438	1.8.E-05
Q12980	GATOR1 complex protein NPRL3	4.435	2.9.E-02
Q8WUY8	Probable N-acetyltransferase 14	4.421	1.2.E-02
P40337	von Hippel-Lindau disease tumor suppressor	4.408	1.2.E-06
O60936	Nucleolar protein 3	4.387	3.6.E-02
Q9Y315	Deoxyribose-phosphate aldolase	4.373	3.4.E-02
P17706	Tyrosine-protein phosphatase non-receptor type 2	4.331	7.9.E-07
P55327	Tumor protein D52	4.330	1.2.E-02
Q6I9Y2	THO complex subunit 7	4.317	3.2.E-02
Q99942	E3 ubiquitin-protein ligase RNF5	4.310	2.0.E-03
P09104	Gamma-enolase	4.300	5.4.E-05
Q9BZL1	Ubiquitin-like protein 5	4.293	9.3.E-06
Q9Y2K5	R3H domain-containing protein 2	4.254	2.9.E-02
Q9BUB4	tRNA-specific adenosine deaminase 1	4.226	4.4.E-02
P17028	Zinc finger protein 24	4.187	1.2.E-05
Q9NX18	Succinate dehydrogenase assembly factor 2, mitochondrial	4.175	1.6.E-05
Q9Y6E2	eIF5-mimic protein 1	4.172	2.8.E-05
Q96HQ2	CDKN2AIP N-terminal-like protein	4.149	1.4.E-02

Q6DD88	Atlastin-3	4.120	2.2.E-02
Q6ZRQ5	Protein MMS22-like	4.113	1.3.E-02
Q5R3I4	Tetratricopeptide repeat protein 38	4.094	5.5.E-04
Q15382	GTP-binding protein Rheb	4.088	4.2.E-02
Q9UPR3	Nonsense-mediated mRNA decay factor SMG5	4.082	1.8.E-02
Q96Q11	CCA tRNA nucleotidyltransferase 1, mitochondrial	4.059	2.3.E-02
Q8IUI8	Cytokine receptor-like factor 3	4.052	2.7.E-02
Q86SK9	Stearoyl-CoA desaturase 5	4.046	2.9.E-02
Q5TAQ9	DDB1- and CUL4-associated factor 8	4.042	5.0.E-03
Q96T60	Bifunctional polynucleotide phosphatase/kinase	4.037	8.1.E-04
O15391	Transcription factor YY2	4.034	4.4.E-03
Q99541	Perilipin-2	4.025	1.3.E-02
Q53H80	Akirin-2	4.013	5.3.E-04
Q5T7W0	Zinc finger protein 618	4.012	1.1.E-02
Q86X55	Histone-arginine methyltransferase CARM1	3.999	9.6.E-07
O43402	ER membrane protein complex subunit 8	3.976	1.7.E-02
P05386	Large ribosomal subunit protein P1	3.972	6.0.E-06
Q96HR9	Receptor expression-enhancing protein 6	3.962	2.7.E-02
Q9Y3D8	Adenylate kinase isoenzyme 6	3.951	1.2.E-04
P42858	Huntingtin	3.950	2.5.E-02
P56545	C-terminal-binding protein 2	3.949	4.9.E-07
Q93015	N-alpha-acetyltransferase 80	3.929	2.1.E-05
Q99551	Transcription termination factor 1, mitochondrial	3.918	3.5.E-03
Q8WXA3	RUN and FYVE domain-containing protein 2	3.915	1.1.E-02
P61020	Ras-related protein Rab-5B	3.891	8.7.E-04
Q9H4G4	Golgi-associated plant pathogenesis-related protein 1	3.863	1.7.E-02
Q9UGQ3	Solute carrier family 2, facilitated glucose transporter member 6	3.836	1.1.E-04
Q15652	Probable JmjC domain-containing histone demethylation protein 2C	3.818	3.2.E-02
P48960	Adhesion G protein-coupled receptor E5	3.816	8.3.E-03
Q8N3D4	EH domain-binding protein 1-like protein 1	3.795	1.3.E-03
P35527	Keratin, type I cytoskeletal 9	3.775	1.4.E-07
A6NDU8	RAB7A-interacting MON1-CCZ1 complex subunit 1	3.760	2.2.E-03
Q9HBF4	Zinc finger FYVE domain-containing protein 1	3.731	5.7.E-03
P19367	Hexokinase-1	3.728	5.1.E-06
Q9UHA2	SS18-like protein 2	3.717	2.1.E-04
Q8IUI4;Q8TEQ0	Putative protein SNX29P2	3.705	3.1.E-06
O00762	Ubiquitin-conjugating enzyme E2 C	3.703	2.3.E-02
Q92973	Transportin-1	3.697	7.3.E-05
O96018	Amyloid-beta A4 precursor protein-binding family A member 3	3.675	1.0.E-02
Q9BXK5	Bcl-2-like protein 13	3.667	3.5.E-02
Q99717	Mothers against decapentaplegic homolog 5	3.655	5.3.E-09
Q8WXG6	MAP kinase-activating death domain protein	3.655	1.7.E-02
P09525	Annexin A4	3.653	2.4.E-02
Q96MW5	Conserved oligomeric Golgi complex subunit 8	3.643	1.1.E-03
Q13794	Phorbol-12-myristate-13-acetate-induced protein 1	3.633	5.5.E-03
Q92522	Histone H1.10	3.632	5.8.E-05
Q07020	Large ribosomal subunit protein eL18	3.624	7.6.E-09
Q9HC77	Centrosomal P4.1-associated protein	3.610	1.9.E-02
Q1ZZU3	DNA repair protein SWI5 homolog	3.602	4.2.E-06
P63151	Serine/threonine-protein phosphatase 2A 55 kDa regulatory subunit B alpha isoform	3.601	1.6.E-08
P54727	UV excision repair protein RAD23 homolog B	3.587	7.4.E-07
P55060	Exportin-2	3.568	3.7.E-07
P40426	Pre-B-cell leukemia transcription factor 3	3.568	2.3.E-02
Q9H7B4	Histone-lysine N-methyltransferase SMYD3	3.555	9.3.E-03
Q7Z6B7	SLIT-ROBO Rho GTPase-activating protein 1	3.541	8.5.E-03
Q7Z7N9	Transmembrane protein 179B	3.537	2.7.E-03
O95273	Cyclin-D1-binding protein 1	3.506	3.8.E-02
Q8TDZ2	[F-actin]-monooxygenase MICAL1	3.502	8.2.E-03
Q96FH0	BLOC-1-related complex subunit 8	3.500	2.1.E-03
Q8NCN2	Zinc finger and BTB domain-containing protein 34	3.492	1.5.E-03
Q9H081	Protein MIS12 homolog	3.490	2.7.E-03
Q9BWL3	Protein C1orf43	3.459	7.7.E-03
Q9Y3S2	Zinc finger protein 330	3.456	2.9.E-07
O94760	N(G),N(G)-dimethylarginine dimethylaminohydrolase 1	3.448	3.3.E-02
P27797	Calreticulin	3.445	1.1.E-04
P31751	RAC-beta serine/threonine-protein kinase	3.439	8.0.E-06
Q96EC8	Protein YIPF6	3.432	3.0.E-05
Q6PJP8	DNA cross-link repair 1A protein	3.430	7.8.E-03
A8MVS5	Protein HIDE1	3.417	2.8.E-02
Q9UK33	Zinc finger protein 580	3.416	2.9.E-03
Q9H0W9	Ester hydrolase C11orf54	3.415	2.2.E-03
P14174	Macrophage migration inhibitory factor	3.412	2.5.E-06
O43716	Glutamyl-tRNA(Gln) amidotransferase subunit C, mitochondrial	3.395	3.3.E-06
Q96AD5	Patatin-like phospholipase domain-containing protein 2	3.384	2.6.E-02
Q8IWD4	Coiled-coil domain-containing protein 117	3.367	8.8.E-03
Q9C0E2	Exportin-4	3.367	1.4.E-06
P07602	Prosaposin	3.351	3.9.E-02
Q9Y330	Zinc finger and BTB domain-containing protein 12	3.344	1.5.E-05
Q96K80	Zinc finger CCCH domain-containing protein 10	3.341	3.2.E-05
O95299	NADH dehydrogenase [ubiquinone] 1 alpha subcomplex subunit 10, mitochondrial	3.337	5.2.E-08
P35610	Sterol O-acyltransferase 1	3.299	3.4.E-05
P61960	Ubiquitin-fold modifier 1	3.293	1.0.E-03
A0A0U1RRE5	Negative regulator of P-body association	3.289	6.2.E-06
P15260	Interferon gamma receptor 1	3.289	1.8.E-03
P49770	Translation initiation factor eIF2B subunit beta	3.276	4.3.E-04
Q16401	26S proteasome non-ATPase regulatory subunit 5	3.275	2.2.E-02
Q9UK59	Lariat debranching enzyme	3.266	3.9.E-04
Q9UHJ6	Sedoheptulokinase	3.251	1.4.E-02

P02787	Serotransferrin	3.246	7.0.E-03
Q08AH3;Q68CK6	Acyl-coenzyme A synthetase ACSM2A, mitochondrial	3.238	7.0.E-05
P56178;Q07687	Homeobox protein DLX-5	3.228	3.8.E-04
Q8NI22	Multiple coagulation factor deficiency protein 2	3.217	2.2.E-02
Q02978	Mitochondrial 2-oxoglutarate/malate carrier protein	3.217	1.2.E-02
Q9BYM8	RanBP-type and C3HC4-type zinc finger-containing protein 1	3.209	1.7.E-03
Q6NUQ1	RAD50-interacting protein 1	3.192	5.8.E-04
P35241	Radixin	3.155	4.5.E-09
Q8WUH6	Transmembrane protein 263	3.136	6.5.E-08
Q14CX7	N-alpha-acetyltransferase 25, NatB auxiliary subunit	3.132	3.3.E-02
Q9NVR2	Integrator complex subunit 10	3.117	3.8.E-02
Q8N5L8	Ribonuclease P protein subunit p25-like protein	3.104	1.9.E-06
Q9NVM6	DnaJ homolog subfamily C member 17	3.100	1.1.E-05
O14879	Interferon-induced protein with tetratricopeptide repeats 3	3.092	1.7.E-04
P46976	Glycogenin-1	3.087	1.6.E-02
Q6P1Q9	tRNA N(3)-cytidine methyltransferase METTL2B	3.082	2.7.E-04
15198;P84022;Q157	Mothers against decapentaplegic homolog 9	3.080	5.9.E-06
P16278	Beta-galactosidase	3.074	3.2.E-02
Q06481	Amyloid beta precursor like protein 2	3.068	4.9.E-02
Q9BV81	ER membrane protein complex subunit 6	3.062	1.9.E-02
Q8N5M1	ATP synthase mitochondrial F1 complex assembly factor 2	3.060	1.8.E-02
Q9UID6	Zinc finger protein 639	3.057	7.4.E-05
Q9H0K1	Serine/threonine-protein kinase SIK2	3.056	4.5.E-02
P30049	ATP synthase subunit delta, mitochondrial	3.053	1.1.E-06
Q8IVT5	Kinase suppressor of Ras 1	3.050	1.9.E-05
P20645	Cation-dependent mannose-6-phosphate receptor	3.046	1.0.E-04
Q9BQB6	Vitamin K epoxide reductase complex subunit 1	3.043	2.5.E-04
P07311	Acylphosphatase-1	3.043	9.4.E-03
O14924	Regulator of G-protein signaling 12	3.042	3.0.E-02
Q05193	Dynamin-1	3.041	1.9.E-02
P00918	Carbonic anhydrase 2	3.039	6.4.E-03
Q71UM5	Ribosomal protein eS27-like	3.031	3.6.E-02
P41091	Eukaryotic translation initiation factor 2 subunit 3	3.028	1.0.E-03
P56378	ATP synthase subunit ATP5MJ, mitochondrial	3.025	2.1.E-02
O75909	Cyclin-K	3.022	7.2.E-05
O75157	TSC22 domain family protein 2	3.016	5.0.E-05
Q8TBE9	N-acylneuraminate-9-phosphatase	3.014	2.6.E-02
O60291	E3 ubiquitin-protein ligase MGRN1	3.007	3.3.E-02
O95178	NADH dehydrogenase [ubiquinone] 1 beta subcomplex subunit 2, mitochondrial	3.006	6.7.E-06
Q9H4A6	Golgi phosphoprotein 3	2.988	4.2.E-02
P08047	Transcription factor Sp1	2.985	2.2.E-03
O14727	Apoptotic protease-activating factor 1	2.985	2.7.E-04
Q9P0B6	Coiled-coil domain-containing protein 167	2.965	2.5.E-02
Q8WZA0	Protein LZIC	2.956	5.1.E-04
Q9BTC8	Metastasis-associated protein MTA3	2.956	4.7.E-05
Q99871	HAUS augmin-like complex subunit 7	2.952	5.8.E-05
P05387	Large ribosomal subunit protein P2	2.948	1.7.E-08
Q5VV52	Zinc finger protein 691	2.942	4.0.E-02
Q9UI95	Mitotic spindle assembly checkpoint protein MAD2B	2.940	1.3.E-07
Q9NUA8	Zinc finger and BTB domain-containing protein 40	2.940	2.6.E-02
Q4VC31	Protein MIX23	2.924	2.6.E-02
P62942	Peptidyl-prolyl cis-trans isomerase FKBP1A	2.921	3.6.E-06
P67870	Casein kinase II subunit beta	2.915	1.2.E-04
Q9HD34	LYR motif-containing protein 4	2.913	3.1.E-03
P04637	Cellular tumor antigen p53	2.908	1.7.E-03
Q9Y3C4	EKC/KEOPS complex subunit TPRKB	2.901	1.4.E-07
P31939	Bifunctional purine biosynthesis protein ATIC	2.900	2.9.E-06
P45954	Short/branched chain specific acyl-CoA dehydrogenase, mitochondrial	2.894	5.1.E-04
Q6ZU35	Capping protein-inhibiting regulator of actin dynamics	2.881	5.7.E-06
Q9HCC0	Methylcrotonoyl-CoA carboxylase beta chain, mitochondrial	2.880	1.4.E-05
Q92536	Y+L amino acid transporter 2	2.879	2.2.E-06
Q9H0A8	COMM domain-containing protein 4	2.875	4.3.E-02
Q5VYK3	Proteasome adapter and scaffold protein ECM29	2.863	6.2.E-07
Q9BUL8	Programmed cell death protein 10	2.854	6.6.E-05
Q4U2R6	Large ribosomal subunit protein mL51	2.850	8.7.E-05
Q8IWL3	Iron-sulfur cluster co-chaperone protein HscB	2.847	1.1.E-03
P30153	Serine/threonine-protein phosphatase 2A 65 kDa regulatory subunit A alpha isoform	2.846	4.6.E-08
P63272	Transcription elongation factor SPT4	2.844	2.3.E-06
O15371	Eukaryotic translation initiation factor 3 subunit D	2.834	3.5.E-06
Q9HBM0	Vezatin	2.822	2.4.E-02
Q6NXT1	Ankyrin repeat domain-containing protein 54	2.820	1.9.E-05
Q8NFQ8	Torsin-1A-interacting protein 2	2.812	2.2.E-04
O94813	Slit homolog 2 protein	2.811	5.5.E-03
O15360	Fanconi anemia group A protein	2.806	1.4.E-02
Q9HB66	Alternative protein MKKS	2.800	3.6.E-02
Q92990	Glomulin	2.794	3.4.E-02
Q9Y3C0	WASH complex subunit 3	2.790	1.8.E-02
Q9Y2Z9	Ubiquinone biosynthesis monooxygenase COQ6, mitochondrial	2.790	3.4.E-07
Q99808	Equilibrative nucleoside transporter 1	2.789	8.9.E-04
Q9Y4D1	Disheveled-associated activator of morphogenesis 1	2.783	1.1.E-04
Q9H7P9	Pleckstrin homology domain-containing family G member 2	2.775	1.9.E-02
Q8N344	Mesoderm induction early response protein 2	2.771	2.0.E-03
P12074	Cytochrome c oxidase subunit 6A1, mitochondrial	2.770	9.9.E-05
Q9H999	Pantothenate kinase 3	2.767	3.1.E-02
Q92604	Acyl-CoA:lysophosphatidylglycerol acyltransferase 1	2.765	4.4.E-02
Q01650	Large neutral amino acids transporter small subunit 1	2.761	2.4.E-05
P47712	Cytosolic phospholipase A2	2.743	5.5.E-05
Q96IK1	Biorientation of chromosomes in cell division protein 1	2.740	7.5.E-06

Q9H2U2	Inorganic pyrophosphatase 2, mitochondrial	2.735	1.2.E-08
Q16143	Beta-synuclein	2.734	8.6.E-05
O43676	NADH dehydrogenase [ubiquinone] 1 beta subcomplex subunit 3	2.733	2.0.E-04
P98172	Ephrin-B1	2.732	3.5.E-06
Q13490	Baculoviral IAP repeat-containing protein 2	2.728	2.0.E-04
Q7RTR2	NLR family CARD domain-containing protein 3	2.724	6.5.E-06
P57678	Gem-associated protein 4	2.720	1.0.E-03
Q16342	Programmed cell death protein 2	2.715	5.0.E-05
Q96IY1	Kinetochore-associated protein NSL1 homolog	2.714	3.2.E-06
Q9HAD4	WD repeat-containing protein 41	2.711	7.2.E-07
Q13445	Transmembrane emp24 domain-containing protein 1	2.710	8.0.E-03
Q96QF0	Rab-3A-interacting protein	2.709	1.4.E-02
Q6UWI4	Protein shisa-2 homolog	2.707	4.7.E-02
Q01081	Splicing factor U2AF 35 kDa subunit	2.700	4.6.E-05
Q66K14	TBC1 domain family member 9B	2.698	3.4.E-02
O60573	Eukaryotic translation initiation factor 4E type 2	2.696	2.3.E-04
Q96ER9	Mitochondrial potassium channel	2.692	7.8.E-06
Q9H9J2	Large ribosomal subunit protein mL44	2.691	1.9.E-03
P13645	Keratin, type I cytoskeletal 10	2.689	2.6.E-06
Q9GZR7	ATP-dependent RNA helicase DDX24	2.688	9.0.E-04
Q96QK1	Vacuolar protein sorting-associated protein 35	2.683	4.7.E-05
Q6P1R4	tRNA-dihydrouridine(16/17) synthase [NAD(P)(+)]-like	2.682	5.9.E-06
P07949	Proto-oncogene tyrosine-protein kinase receptor Ret	2.677	6.4.E-05
P14209	CD99 antigen	2.677	6.1.E-03
O94910	Adhesion G protein-coupled receptor L1	2.675	4.5.E-03
DMV0;P0DMV1;P0DI	Cancer/testis antigen family 45 member A6	2.674	2.0.E-03
Q9UBB6	Neurochondrin	2.672	2.7.E-04
Q13637	Ras-related protein Rab-32	2.671	3.4.E-03
Q08379	Golgin subfamily A member 2	2.671	4.9.E-04
P11387	DNA topoisomerase 1	2.670	1.4.E-04
P40937	Replication factor C subunit 5	2.669	1.3.E-05
O00592	Podocalyxin	2.667	2.3.E-05
Q15650	Activating signal cointegrator 1	2.667	2.5.E-06
O95302;Q75LS8	Peptidyl-prolyl cis-trans isomerase FKBP9	2.665	1.3.E-04
Q9H583	HEAT repeat-containing protein 1	2.663	1.6.E-05
P54725	UV excision repair protein RAD23 homolog A	2.658	1.6.E-05
P09234	U1 small nuclear ribonucleoprotein C	2.658	7.8.E-07
A8MZ97	Uncharacterized protein C2orf74	2.655	3.0.E-03
P52569	Cationic amino acid transporter 2	2.646	1.2.E-03
Q96CN4	EVI5-like protein	2.645	5.1.E-05
Q06323	Proteasome activator complex subunit 1	2.644	8.3.E-07
Q7L2H7	Eukaryotic translation initiation factor 3 subunit M	2.644	8.5.E-03
Q9BVC6	Voltage-gated monoatomic cation channel TMEM109	2.642	2.1.E-02
Q9H814	Phosphorylated adapter RNA export protein	2.642	2.6.E-07
Q5JVF3	PCI domain-containing protein 2	2.640	4.4.E-02
P53701	Holocytochrome c-type synthase	2.632	1.1.E-03
O95202	Mitochondrial proton/calcium exchanger protein	2.629	3.7.E-08
Q9UIU6	Homeobox protein SIX4	2.628	2.1.E-05
Q68CQ4	U3 small nucleolar RNA-associated protein 25 homolog	2.625	2.8.E-06
O43156	TELO2-interacting protein 1 homolog	2.623	2.3.E-02
Q96S94	Cyclin-L2	2.618	3.6.E-06
P14678	Small nuclear ribonucleoprotein-associated proteins B and B'	2.617	2.2.E-07
Q96N67	Dedicator of cytokinesis protein 7	2.616	1.3.E-06
Q69YN4	Protein virilizer homolog	2.614	1.6.E-05
Q9NUD5	Zinc finger CCHC domain-containing protein 3	2.614	1.4.E-05
O95602	DNA-directed RNA polymerase I subunit RPA1	2.609	3.8.E-03
P52758	2-iminobutanoate/2-iminopropanoate deaminase	2.604	4.6.E-02
P28702	Retinoic acid receptor RXR-beta	2.599	6.1.E-05
Q8TB36	Ganglioside-induced differentiation-associated protein 1	2.596	3.6.E-02
Q9NNX1	Tuftelin	2.592	2.9.E-04
Q8IY22	C-Maf-inducing protein	2.590	2.4.E-02
Q9BW66	Cyclin-dependent kinase 2-interacting protein	2.589	3.5.E-04
O14925	Mitochondrial import inner membrane translocase subunit Tim23	2.587	4.3.E-06
P56962	Syntaxin-17	2.587	1.3.E-04
Q9H3R5	Centromere protein H	2.583	1.8.E-03
Q01814	Plasma membrane calcium-transporting ATPase 2	2.581	1.5.E-05
O14733	Dual specificity mitogen-activated protein kinase kinase 7	2.577	2.1.E-05
Q96QD8	Sodium-coupled neutral amino acid symporter 2	2.575	1.6.E-06
P61353	Large ribosomal subunit protein eL27	2.574	3.1.E-02
P60510	Serine/threonine-protein phosphatase 4 catalytic subunit	2.571	5.3.E-04
Q96B01	RAD51-associated protein 1	2.568	9.2.E-05
Q96J01	THO complex subunit 3	2.568	7.2.E-05
P63167	Dynein light chain 1, cytoplasmic	2.568	8.0.E-03
O43379	WD repeat-containing protein 62	2.568	3.2.E-09
Q9Y2V7	Conserved oligomeric Golgi complex subunit 6	2.566	3.6.E-03
Q96T88	E3 ubiquitin-protein ligase UHRF1	2.565	6.0.E-04
Q9H9R9	Dysbindin domain-containing protein 1	2.561	1.3.E-03
P09211	Glutathione S-transferase P	2.559	5.4.E-05
Q9H3H3	UPF0696 protein C11orf68	2.552	5.1.E-04
Q9BV86	N-terminal Xaa-Pro-Lys N-methyltransferase 1	2.549	1.0.E-04
Q9UP83	Conserved oligomeric Golgi complex subunit 5	2.549	8.5.E-05
P04264	Keratin, type II cytoskeletal 1	2.542	4.2.E-05
Q9UKI2	Cdc42 effector protein 3	2.535	3.5.E-03
O00458	Interferon-related developmental regulator 1	2.528	7.0.E-03
Q8NDT2	Putative RNA-binding protein 15B	2.526	2.7.E-05
Q8N1I0	Dedicator of cytokinesis protein 4	2.520	6.0.E-04
Q6P3X3	Tetratricopeptide repeat protein 27	2.518	1.5.E-05
Q9NX58	Cell growth-regulating nucleolar protein	2.518	2.5.E-04

Q5VIR6	Vacuolar protein sorting-associated protein 53 homolog	2.518	3.9.E-04
O60831	PRA1 family protein 2	2.515	2.4.E-04
P52815	Large ribosomal subunit protein bL12m	2.514	5.6.E-06
Q14997	Proteasome activator complex subunit 4	2.511	7.4.E-03
Q9BZK7	F-box-like/WD repeat-containing protein TBL1XR1	2.510	1.5.E-04
Q9H8Y8	Golgi reassembly-stacking protein 2	2.509	1.8.E-05
Q9BWS9	Chitinase domain-containing protein 1	2.507	3.5.E-05
O60888	Protein CutA	2.504	4.2.E-03
C9J7I0	UBAP1-MVB12-associated (UMA)-domain containing protein 1	2.503	2.1.E-03
Q14790	Caspase-8	2.502	8.4.E-06
Q9Y4E8	Ubiquitin carboxyl-terminal hydrolase 15	2.500	8.8.E-06
Q9NX05	Constitutive coactivator of PPAR-gamma-like protein 2	2.496	3.3.E-05
Q8NHH9	Atlastin-2	2.495	3.6.E-03
Q9BWJ5	Splicing factor 3B subunit 5	2.493	9.1.E-07
P19622	Homeobox protein engrailed-2	2.492	1.9.E-05
O95777	U6 snRNA-associated Sm-like protein LSm8	2.489	4.8.E-02
P36404	ADP-ribosylation factor-like protein 2	2.485	9.3.E-05
Q6NXE6	Armadillo repeat-containing protein 6	2.485	7.9.E-08
P62318	Small nuclear ribonucleoprotein Sm D3	2.484	3.0.E-07
Q9Y3P4	Rhomboid domain-containing protein 3	2.482	4.0.E-04
P06280	Alpha-galactosidase A	2.482	3.3.E-05
Q86Y82	Syntaxin-12	2.482	8.3.E-04
Q96HE7	ERO1-like protein alpha	2.481	4.8.E-02
P25787	Proteasome subunit alpha type-2	2.475	3.4.E-05
Q06203	Amidophosphoribosyltransferase	2.473	1.6.E-06
P58546	Myotrophin	2.471	4.5.E-04
P19623	Spermidine synthase	2.469	5.3.E-05
Q9NP31	SH2 domain-containing protein 2A	2.467	2.6.E-03
P42345	Serine/threonine-protein kinase mTOR	2.467	5.8.E-04
Q13162	Peroxiredoxin-4	2.458	1.2.E-07
)DP23;P0DP24;P0DP	Calmodulin-1	2.457	6.3.E-06
Q9H3U1	Protein unc-45 homolog A	2.457	9.5.E-04
Q96NX9	Dachshund homolog 2	2.456	1.5.E-03
Q9HCI6	E3 SUMO-protein ligase KIAA1586	2.455	1.9.E-04
Q9NVU0	DNA-directed RNA polymerase III subunit RPC5	2.452	4.9.E-07
P26885	Peptidyl-prolyl cis-trans isomerase FKBP2	2.450	3.0.E-03
O75175	CCR4-NOT transcription complex subunit 3	2.448	1.3.E-07
Q9BRA2	Thioredoxin domain-containing protein 17	2.443	9.8.E-06
P42704	Leucine-rich PPR motif-containing protein, mitochondrial	2.443	7.2.E-12
P50851	Lipopolysaccharide-responsive and beige-like anchor protein	2.441	1.0.E-04
P02533	Keratin, type I cytoskeletal 14	2.440	4.5.E-04
Q53GQ0	Very-long-chain 3-oxoacyl-CoA reductase	2.439	7.4.E-07
Q9UBF8	Phosphatidylinositol 4-kinase beta	2.438	7.5.E-04
A5D8V6	Vacuolar protein sorting-associated protein 37C	2.438	3.5.E-05
Q92831	Histone acetyltransferase KAT2B	2.437	2.9.E-03
Q02750	Dual specificity mitogen-activated protein kinase kinase 1	2.435	9.3.E-04
Q6PI98	INO80 complex subunit C	2.432	8.7.E-06
P83876	Thioredoxin-like protein 4A	2.426	2.2.E-06
Q7Z6L1	Tectonin beta-propeller repeat-containing protein 1	2.424	2.8.E-05
P51948	CDK-activating kinase assembly factor MAT1	2.423	3.7.E-07
Q9NXE8	Pre-mRNA-splicing factor CWC25 homolog	2.423	6.2.E-08
Q9BUI4	DNA-directed RNA polymerase III subunit RPC3	2.422	1.0.E-02
Q00613	Heat shock factor protein 1	2.422	4.7.E-07
Q9BPX6	Calcium uptake protein 1, mitochondrial	2.422	3.6.E-02
Q9NUP1	Biogenesis of lysosome-related organelles complex 1 subunit 4	2.421	7.1.E-03
Q9Y394	Dehydrogenase/reductase SDR family member 7	2.419	1.1.E-03
Q9Y3A4	Ribosomal RNA-processing protein 7 homolog A	2.418	1.2.E-04
Q8IXW5	Putative RNA polymerase II subunit B1 CTD phosphatase RPAP2	2.416	1.4.E-02
Q15678	Tyrosine-protein phosphatase non-receptor type 14	2.415	4.2.E-08
Q5VZE5	N-alpha-acetyltransferase 35, NatC auxiliary subunit	2.414	1.1.E-06
Q9NWZ8	Gem-associated protein 8	2.413	4.0.E-04
Q9UJW0	Dynactin subunit 4	2.409	1.2.E-05
P10074	Telomere zinc finger-associated protein	2.405	3.6.E-02
Q4VC05	B-cell CLL/lymphoma 7 protein family member A	2.402	2.6.E-04
Q9NQ55	Suppressor of SWI4 1 homolog	2.399	1.1.E-04
Q9H0K6	Pseudouridylate synthase PUS7L	2.399	6.0.E-03
Q8IY17	Patatin-like phospholipase domain-containing protein 6	2.396	1.4.E-02
Q9NWM3	CUE domain-containing protein 1	2.395	1.9.E-02
P50219	Motor neuron and pancreas homeobox protein 1	2.392	1.9.E-04
Q9NQ31	A-kinase-interacting protein 1	2.391	1.9.E-03
Q3MII6	TBC1 domain family member 25	2.390	3.0.E-04
O43765	Small glutamine-rich tetratricopeptide repeat-containing protein alpha	2.389	1.3.E-04
O94788	Retinal dehydrogenase 2	2.379	1.5.E-05
P06576	ATP synthase subunit beta, mitochondrial	2.374	5.5.E-06
P61619;Q9H9S3	Protein transport protein Sec61 subunit alpha isoform 1	2.373	1.4.E-02
P23786	Carnitine O-palmitoyltransferase 2, mitochondrial	2.373	7.3.E-03
Q99622	Protein C10	2.372	1.6.E-03
P61244	Protein max	2.371	2.6.E-07
O14929	Histone acetyltransferase type B catalytic subunit	2.371	8.6.E-04
Q96J42	Thioredoxin domain-containing protein 15	2.370	3.7.E-02
Q8WX92	Negative elongation factor B	2.370	1.8.E-04
Q96AC1	Fermitin family homolog 2	2.366	2.5.E-06
Q9UBD5	Origin recognition complex subunit 3	2.365	1.1.E-04
Q14CB8	Rho GTPase-activating protein 19	2.363	2.0.E-03
Q8NI36	WD repeat-containing protein 36	2.362	6.2.E-07
Q9NVH1	DnaJ homolog subfamily C member 11	2.362	1.4.E-02
Q9Y575	Ankyrin repeat and SOCS box protein 3	2.359	6.7.E-03
Q86W50	RNA N(6)-adenosine-methyltransferase METTL16	2.351	3.3.E-04

Q8IWR0	Zinc finger CCCH domain-containing protein 7A	2.349	1.0.E-06
Q8IVH2	Forkhead box protein P4	2.349	1.9.E-03
Q86X83	COMM domain-containing protein 2	2.349	2.3.E-03
Q13309	S-phase kinase-associated protein 2	2.346	3.5.E-02
Q8N2F6	Armadillo repeat-containing protein 10	2.345	1.1.E-02
O00231	26S proteasome non-ATPase regulatory subunit 11	2.343	3.4.E-05
Q2M1P5	Kinesin-like protein KIF7	2.340	2.8.E-06
Q86V35	Calcium-binding protein 7	2.339	5.2.E-07
P17568	NADH dehydrogenase [ubiquinone] 1 beta subcomplex subunit 7	2.338	7.9.E-06
Q7L8W6	Diphthine--ammonia ligase	2.338	1.3.E-02
P50897	Palmitoyl-protein thioesterase 1	2.336	7.3.E-04
Q6DKJ4	Nucleoredoxin	2.335	2.9.E-02
O43542	DNA repair protein XRCC3	2.334	9.6.E-04
Q96A35	Large ribosomal subunit protein uL24m	2.333	3.7.E-05
Q9UGY1	Nucleolar protein 12	2.331	2.8.E-04
Q96GD4	Aurora kinase B	2.327	6.9.E-07
Q9BVM2	Protein DPCD	2.321	4.6.E-02
Q86VP6	Cullin-associated NEDD8-dissociated protein 1	2.318	9.0.E-04
P19404	NADH dehydrogenase [ubiquinone] flavoprotein 2, mitochondrial	2.318	3.0.E-03
P49005	DNA polymerase delta subunit 2	2.316	4.4.E-07
P61088	Ubiquitin-conjugating enzyme E2 N	2.316	1.4.E-05
Q06124	Tyrosine-protein phosphatase non-receptor type 11	2.316	2.5.E-07
O14613	Cdc42 effector protein 2	2.316	7.8.E-04
P61966	AP-1 complex subunit sigma-1A	2.315	6.6.E-07
Q9BQF6	Sentrin-specific protease 7	2.314	1.7.E-04
O75071	EF-hand calcium-binding domain-containing protein 14	2.312	1.9.E-03
Q15257	Serine/threonine-protein phosphatase 2A activator	2.311	2.7.E-02
Q8N131	Porimin	2.307	9.5.E-03
Q6PCD5	E3 ubiquitin-protein ligase RFWD3	2.306	1.7.E-04
Q5U5X0	Complex III assembly factor LYRM7	2.304	1.6.E-04
Q86VI3	Ras GTPase-activating-like protein IQGAP3	2.304	3.9.E-02
Q99598	Translin-associated protein X	2.302	7.1.E-04
P13798	Acylamino-acid-releasing enzyme	2.302	1.9.E-05
P42766	Large ribosomal subunit protein uL29	2.300	1.2.E-06
Q9NPA8	Transcription and mRNA export factor ENY2	2.300	4.7.E-05
P62714;P67775	Serine/threonine-protein phosphatase 2A catalytic subunit beta isoform	2.298	2.8.E-08
O15127	Secretory carrier-associated membrane protein 2	2.297	3.8.E-06
Q9BRP1	Programmed cell death protein 2-like	2.292	5.9.E-07
Q15390	Mitochondrial fission regulator 1	2.292	6.2.E-04
Q5J8M3	ER membrane protein complex subunit 4	2.292	2.6.E-04
Q8N4S9	MARVEL domain-containing protein 2	2.290	6.8.E-03
Q9Y2Z2	Protein MTO1 homolog, mitochondrial	2.287	6.1.E-04
O75127	Pentatricopeptide repeat-containing protein 1, mitochondrial	2.286	2.1.E-03
Q9Y5Z7	Host cell factor 2	2.286	3.4.E-02
Q9BWK5	Cell cycle regulator of non-homologous end joining	2.286	3.5.E-05
Q8WUW1	Protein BRICK1	2.284	2.1.E-05
Q15436	Protein transport protein Sec23A	2.284	7.3.E-05
Q9GZN2	Homeobox protein TGIF2	2.283	1.2.E-02
Q658P3	Metalloreductase STEAP3	2.282	1.4.E-02
Q07812	Apoptosis regulator BAX	2.282	4.1.E-07
P12532	Creatine kinase U-type, mitochondrial	2.282	1.3.E-04
P20936	Ras GTPase-activating protein 1	2.278	2.3.E-02
P52565	Rho GDP-dissociation inhibitor 1	2.274	3.0.E-05
Q9BPZ3	Polyadenylate-binding protein-interacting protein 2	2.274	2.6.E-06
P56182	Ribosomal RNA processing protein 1 homolog A	2.274	7.6.E-05
Q9H8H0	Nucleolar protein 11	2.272	1.5.E-05
Q9UNP9	Peptidyl-prolyl cis-trans isomerase E	2.272	2.5.E-06
Q9H1I8	Activating signal cointegrator 1 complex subunit 2	2.272	2.0.E-08
Q86XN8	RNA-binding protein MEX3D	2.270	2.6.E-05
Q6UWE0	E3 ubiquitin-protein ligase LRSAM1	2.264	2.0.E-07
P50990	T-complex protein 1 subunit theta	2.263	8.4.E-09
Q9H9P5	Putative E3 ubiquitin-protein ligase UNKL	2.262	1.3.E-02
Q9BUW7	Bublin coiled-coil protein	2.258	2.5.E-03
Q9BVI0	PHD finger protein 20	2.255	6.3.E-05
Q9UPT5	Exocyst complex component 7	2.254	6.5.E-08
Q96AQ6	Pre-B-cell leukemia transcription factor-interacting protein 1	2.252	2.2.E-04
P78362	SRSF protein kinase 2	2.250	1.8.E-02
P18859	ATP synthase-coupling factor 6, mitochondrial	2.249	4.1.E-08
Q9Y6D5	Brefeldin A-inhibited guanine nucleotide-exchange protein 2	2.247	2.1.E-06
Q15428	Splicing factor 3A subunit 2	2.244	1.0.E-06
Q9UGJ1	Gamma-tubulin complex component 4	2.242	4.8.E-04
Q96SU4	Oxysterol-binding protein-related protein 9	2.241	2.1.E-05
P55199	RNA polymerase II elongation factor ELL	2.241	2.1.E-08
Q9BSV6	tRNA-splicing endonuclease subunit Sen34	2.241	1.1.E-06
P98179	RNA-binding protein 3	2.240	2.5.E-07
Q07065	Cytoskeleton-associated protein 4	2.240	1.8.E-05
Q9Y2Q3	Glutathione S-transferase kappa 1	2.236	6.4.E-04
P51946	Cyclin-H	2.235	2.7.E-06
Q9NUJ3	T-complex protein 11-like protein 1	2.235	9.9.E-04
Q8N302	Angiogenic factor with G patch and FHA domains 1	2.234	1.2.E-02
Q9HB09	Bcl-2-like protein 12	2.234	2.0.E-03
Q9UL46	Proteasome activator complex subunit 2	2.227	6.9.E-03
Q9HC07	Putative divalent cation/proton antiporter TMEM165	2.224	2.6.E-03
Q5JTW2	Centrosomal protein of 78 kDa	2.222	4.6.E-02
Q9H3G5	Probable serine carboxypeptidase CPVL	2.220	1.2.E-06
Q01658	Protein Dr1	2.219	6.7.E-07
Q12959	Disks large homolog 1	2.216	3.2.E-04
O43399	Tumor protein D54	2.215	1.0.E-06

Q96P48	Arf-GAP with Rho-GAP domain, ANK repeat and PH domain-containing protein 1	2.215	3.0.E-05
O60256	Phosphoribosyl pyrophosphate synthase-associated protein 2	2.214	1.6.E-09
Q86V21	Acetoacetyl-CoA synthetase	2.213	3.0.E-03
Q9NVS2	Large ribosomal subunit protein mL66	2.211	3.1.E-05
P55268	Laminin subunit beta-2	2.210	1.7.E-02
O76021	Ribosomal L1 domain-containing protein 1	2.210	1.1.E-04
Q9UFF9	CCR4-NOT transcription complex subunit 8	2.208	5.1.E-03
Q15121	Astrocytic phosphoprotein PEA-15	2.201	1.3.E-04
Q92688	Acidic leucine-rich nuclear phosphoprotein 32 family member B	2.201	4.1.E-04
Q9H6E5	Speckle targeted PIP5K1A-regulated poly(A) polymerase	2.200	3.4.E-02
Q15843	Ubiquitin-like protein NEDD8	2.199	7.3.E-06
Q68DQ2	Very large A-kinase anchor protein	2.199	6.7.E-03
Q9BTW9	Tubulin-specific chaperone D	2.198	1.8.E-02
O00161	Synaptosomal-associated protein 23	2.198	6.9.E-03
Q14318	Peptidyl-prolyl cis-trans isomerase FKBP8	2.197	5.2.E-04
Q9H0H0	Integrator complex subunit 2	2.194	2.4.E-02
Q9C0I1	Myotubularin-related protein 12	2.193	2.3.E-04
Q9Y512	Sorting and assembly machinery component 50 homolog	2.193	1.3.E-02
Q9BW83	Intraflagellar transport protein 27 homolog	2.192	5.7.E-05
P61326;Q96A72	Protein mago nashi homolog	2.191	5.4.E-04
P27635	Large ribosomal subunit protein uL16	2.189	4.8.E-06
Q8IXT5	RNA-binding protein 12B	2.188	2.2.E-04
Q9NRF8	CTP synthase 2	2.188	2.3.E-06
O43504	Ragulator complex protein LAMTOR5	2.187	1.1.E-04
O43488	Aflatoxin B1 aldehyde reductase member 2	2.187	2.2.E-06
O95873	Uncharacterized protein C6orf47	2.184	4.2.E-05
Q9H2C2	Protein ARV1	2.182	2.2.E-03
O15126	Secretory carrier-associated membrane protein 1	2.180	2.3.E-04
Q02040	A-kinase anchor protein 17A	2.180	2.1.E-03
Q8NBF2	NHL repeat-containing protein 2	2.179	5.6.E-04
Q8IYB3	Serine/arginine repetitive matrix protein 1	2.175	5.8.E-05
P0C0S5;Q71UI9	Histone H2A.Z	2.174	4.9.E-05
O15260	Surfeit locus protein 4	2.174	5.3.E-04
O00499	Myc box-dependent-interacting protein 1	2.174	3.0.E-04
Q9NX76	CKLF-like MARVEL transmembrane domain-containing protein 6	2.173	4.8.E-02
Q96SB4	SRSF protein kinase 1	2.173	5.2.E-04
Q9UMX1	Suppressor of fused homolog	2.173	2.5.E-06
P46781	Small ribosomal subunit protein uS4	2.172	5.9.E-08
Q8NI37	Protein phosphatase PTC7 homolog	2.171	2.2.E-03
O94804	Serine/threonine-protein kinase 10	2.170	7.2.E-04
P49795	Regulator of G-protein signaling 19	2.168	4.8.E-02
A7E2V4	Zinc finger SWIM domain-containing protein 8	2.168	8.1.E-04
Q9Y467	Sal-like protein 2	2.167	8.1.E-05
Q9NWB6	Arginine and glutamate-rich protein 1	2.165	4.7.E-06
Q86TB9	Protein PAT1 homolog 1	2.164	1.1.E-05
Q13576	Ras GTPase-activating-like protein IQGAP2	2.164	3.0.E-04
Q14192	Four and a half LIM domains protein 2	2.164	2.4.E-02
P61289	Proteasome activator complex subunit 3	2.163	2.8.E-08
P12694	2-oxoisovalerate dehydrogenase subunit alpha, mitochondrial	2.161	2.0.E-06
Q9Y4L5	E3 ubiquitin-protein ligase RNF115	2.160	1.2.E-02
Q9NY12	H/ACA ribonucleoprotein complex subunit 1	2.160	7.2.E-05
Q9BRV8	Suppressor of IKBKE 1	2.154	1.2.E-04
Q9P0L0	Vesicle-associated membrane protein-associated protein A	2.153	2.6.E-04
O95872	G patch domain and ankyrin repeat-containing protein 1	2.150	3.8.E-03
Q16637	Survival motor neuron protein	2.148	8.1.E-05
O15217	Glutathione S-transferase A4	2.148	3.7.E-02
Q15287	RNA-binding protein with serine-rich domain 1	2.144	4.0.E-06
Q15042	Rab3 GTPase-activating protein catalytic subunit	2.141	7.1.E-05
Q9H0Z9	RNA-binding protein 38	2.138	2.8.E-08
Q8N8R5	Mitochondrial protein C2orf69	2.134	1.6.E-03
P06744	Glucose-6-phosphate isomerase	2.134	3.0.E-02
P33552	Cyclin-dependent kinases regulatory subunit 2	2.133	4.5.E-05
Q99956	Dual specificity protein phosphatase 9	2.132	2.9.E-07
Q0VGL1	Ragulator complex protein LAMTOR4	2.132	2.2.E-06
Q9UMX0	Ubiquilin-1	2.131	3.0.E-05
Q9H2J7	Sodium-dependent neutral amino acid transporter B(0)AT2	2.131	4.0.E-03
Q92616	Stalled ribosome sensor GCN1	2.130	4.8.E-05
Q9NZ45	CDGSH iron-sulfur domain-containing protein 1	2.130	2.0.E-03
P52747	Zinc finger protein 143	2.130	3.7.E-03
Q2M296	Methenyltetrahydrofolate synthase domain-containing protein	2.129	4.3.E-02
Q7Z2Z2	Elongation factor-like GTPase 1	2.129	3.0.E-04
O60664	Perilipin-3	2.128	4.8.E-09
Q9NYJ1	Cytochrome c oxidase assembly factor 4 homolog, mitochondrial	2.126	2.1.E-03
Q9BQE3	Tubulin alpha-1C chain	2.125	1.9.E-06
Q15758	Neutral amino acid transporter B(0)	2.122	5.5.E-05
P30154	Serine/threonine-protein phosphatase 2A 65 kDa regulatory subunit A beta isoform	2.122	4.8.E-05
P48436	Transcription factor SOX-9	2.121	1.8.E-02
Q8N6S5	ADP-ribosylation factor-like protein 6-interacting protein 6	2.120	1.6.E-04
O95486	Protein transport protein Sec24A	2.118	2.7.E-06
Q8NEB9	Phosphatidylinositol 3-kinase catalytic subunit type 3	2.117	3.8.E-06
Q7Z4H3	5'-deoxynucleotidase HDDC2	2.117	6.8.E-06
Q9BS16	Centromere protein K	2.113	1.4.E-02
P51665	26S proteasome non-ATPase regulatory subunit 7	2.113	9.6.E-08
P62330	ADP-ribosylation factor 6	2.111	3.1.E-02
Q6SZW1	NAD(+) hydrolase SARM1	2.111	5.7.E-03
P38398	Breast cancer type 1 susceptibility protein	2.109	3.9.E-05
O15090	Zinc finger protein 536	2.106	3.0.E-03
P20674	Cytochrome c oxidase subunit 5A, mitochondrial	2.104	7.0.E-05

Q8NFP9	Neurobeachin	2.102	2.5.E-04
A4D1P6	WD repeat-containing protein 91	2.096	5.4.E-06
O75368	Adapter SH3BGRL	2.095	5.3.E-03
Q9UBQ5	Eukaryotic translation initiation factor 3 subunit K	2.095	3.9.E-06
Q32MZ4	Leucine-rich repeat flightless-interacting protein 1	2.094	1.1.E-08
P07919	Cytochrome b-c1 complex subunit 6, mitochondrial	2.091	7.8.E-07
Q32NC0	UPF0711 protein C18orf21	2.090	4.8.E-04
P00390	Glutathione reductase, mitochondrial	2.089	3.6.E-02
P49755	Transmembrane emp24 domain-containing protein 10	2.088	7.4.E-05
Q9UJA5	tRNA (adenine(58)-N(1))-methyltransferase non-catalytic subunit TRM6	2.086	1.2.E-04
Q08AM6	Protein VAC14 homolog	2.084	6.1.E-05
P11047	Laminin subunit gamma-1	2.082	2.2.E-04
Q9C010	cAMP-dependent protein kinase inhibitor beta	2.082	3.8.E-05
O43264	Centromere/kinetochore protein zw10 homolog	2.081	7.1.E-05
P51513	RNA-binding protein Nova-1	2.080	3.2.E-04
Q969T4	Ubiquitin-conjugating enzyme E2 E3	2.080	4.3.E-02
Q9H4B6	Protein salvador homolog 1	2.079	3.8.E-06
O14647	Chromodomain-helicase-DNA-binding protein 2	2.078	9.8.E-03
Q9BVK6	Transmembrane emp24 domain-containing protein 9	2.078	3.4.E-06
Q13641	Trophoblast glycoprotein	2.077	3.6.E-02
Q9Y4P8	WD repeat domain phosphoinositide-interacting protein 2	2.077	4.4.E-02
P11413	Glucose-6-phosphate 1-dehydrogenase	2.076	5.1.E-07
Q8N6N3	UPF0690 protein C1orf52	2.076	9.3.E-07
O43633	Charged multivesicular body protein 2a	2.074	8.7.E-07
Q9H089	Large subunit GTPase 1 homolog	2.073	7.1.E-06
Q92538	Golgi-specific brefeldin A-resistance guanine nucleotide exchange factor 1	2.071	6.5.E-04
Q8TDH9	Biogenesis of lysosome-related organelles complex 1 subunit 5	2.071	1.7.E-04
Q6IEG0	U11/U12 small nuclear ribonucleoprotein 48 kDa protein	2.071	2.4.E-04
Q96A65	Exocyst complex component 4	2.070	4.0.E-05
7;Q15561;Q15562;Q	Transcriptional enhancer factor TEF-1	2.069	5.3.E-07
Q9UNW9	RNA-binding protein Nova-2	2.068	5.1.E-04
Q86UP2	Kinectin	2.068	3.0.E-05
P0CG40	Transcription factor Sp9	2.063	2.0.E-04
P78312	Protein FAM193A	2.062	2.9.E-05
Q9H0R6	Glutamyl-tRNA(Gln) amidotransferase subunit A, mitochondrial	2.060	1.2.E-02
Q96JG8	Melanoma-associated antigen D4	2.059	3.9.E-04
Q5H9I0	Transcription factor Dp family member 3	2.058	4.6.E-03
P12236	ADP/ATP translocase 3	2.056	1.0.E-02
P34931	Heat shock 70 kDa protein 1-like	2.055	2.2.E-05
P60900	Proteasome subunit alpha type-6	2.055	1.3.E-03
O95229	Outer kinetochore KNL1 complex subunit ZWINT	2.054	3.1.E-04
Q96DA2	Ras-related protein Rab-39B	2.054	8.7.E-07
Q99963	Endophilin-A3	2.051	3.7.E-04
P05388	Large ribosomal subunit protein uL10	2.051	9.8.E-09
P53350	Serine/threonine-protein kinase PLK1	2.049	2.1.E-06
Q13015	Protein AF1q	2.048	1.0.E-04
Q9P0V9	Septin-10	2.047	1.1.E-03
Q6UXH1	Protein disulfide isomerase CRELD2	2.047	1.4.E-03
Q9Y2Q9	Small ribosomal subunit protein bS1m	2.047	3.7.E-06
Q6ZSY5	Protein phosphatase 1 regulatory subunit 3F	2.044	1.2.E-02
P06241	Tyrosine-protein kinase Fyn	2.043	4.5.E-02
Q99576	TSC22 domain family protein 3	2.042	2.2.E-02
Q96IW7	Vesicle-trafficking protein SEC22a	2.042	3.6.E-02
Q9UNM6	26S proteasome non-ATPase regulatory subunit 13	2.041	3.6.E-04
Q9NXN4	Ganglioside-induced differentiation-associated protein 2	2.041	6.0.E-03
Q8NDH6	Islet cell autoantigen 1-like protein	2.040	4.1.E-03
Q9BYG5	Partitioning defective 6 homolog beta	2.040	1.9.E-06
Q9UHD2	Serine/threonine-protein kinase TBK1	2.040	3.1.E-03
P40424	Pre-B-cell leukemia transcription factor 1	2.039	2.3.E-04
Q9Y2W2	WW domain-binding protein 11	2.039	1.7.E-07
P83731	Large ribosomal subunit protein eL24	2.037	1.5.E-04
Q6IN85	Serine/threonine-protein phosphatase 4 regulatory subunit 3A	2.036	8.1.E-04
Q9Y6C9	Mitochondrial carrier homolog 2	2.035	2.7.E-03
Q9BW85	Splicing factor YJU2	2.034	2.5.E-04
Q96CS2	HAUS augmin-like complex subunit 1	2.030	7.6.E-04
Q12907	Vesicular integral-membrane protein VIP36	2.030	2.1.E-02
P62913	Large ribosomal subunit protein uL5	2.028	2.3.E-06
Q9Y496	Kinesin-like protein KIF3A	2.026	1.6.E-05
P26038	Moesin	2.025	2.3.E-07
Q92621	Nuclear pore complex protein Nup205	2.024	1.5.E-08
P23258;Q9NRH3	Tubulin gamma-1 chain	2.024	1.5.E-06
P82914	Small ribosomal subunit protein uS15m	2.023	1.4.E-02
Q6PGQ7	Protein aurora borealis	2.021	1.8.E-05
P47813	Eukaryotic translation initiation factor 1A, X-chromosomal	2.019	7.1.E-07
O95900	Pseudouridylate synthase TRUB2, mitochondrial	2.019	5.6.E-04
Q96EY4	Translation machinery-associated protein 16	2.018	1.6.E-04
Q9UID3	Vacuolar protein sorting-associated protein 51 homolog	2.018	1.0.E-03
Q9NP70	Ameloblastin	2.018	4.3.E-02
Q9BY43	Charged multivesicular body protein 4a	2.017	3.3.E-06
Q9BYI3	Hyccin	2.016	3.0.E-07
Q13029	PR domain zinc finger protein 2	2.015	4.5.E-04
Q9H1H9	Kinesin-like protein KIF13A	2.014	2.1.E-04
O60216	Double-strand-break repair protein rad21 homolog	2.013	3.7.E-07
Q9Y2W6	Tudor and KH domain-containing protein	2.012	8.2.E-05
Q01469	Fatty acid-binding protein 5	2.012	5.0.E-03
A0A2Z4LIS9	Forkhead box protein O3B	2.010	6.8.E-07
O14653	Golgi SNAP receptor complex member 2	2.009	1.7.E-03
O43865	S-adenosylhomocysteine hydrolase-like protein 1	2.008	1.6.E-08

Q9H7L9	Sin3 histone deacetylase corepressor complex component SDS3	2.007	1.2.E-06
Q8IYD1	Eukaryotic peptide chain release factor GTP-binding subunit ERF3B	2.007	1.1.E-03
P51572	B-cell receptor-associated protein 31	2.007	5.3.E-03
Q13601	KRR1 small subunit processome component homolog	2.006	2.0.E-03
Q14137	Ribosome biogenesis protein BOP1	2.005	1.2.E-06
P84098	Large ribosomal subunit protein eL19	2.005	1.1.E-09
Q9NZN5	Rho guanine nucleotide exchange factor 12	2.004	2.0.E-05
P13747	HLA class I histocompatibility antigen, alpha chain E	2.004	8.9.E-04
Q8WW01	tRNA-splicing endonuclease subunit Sen15	2.003	1.8.E-05
Q00765	Receptor expression-enhancing protein 5	2.003	1.3.E-03
P19793	Retinoic acid receptor RXR-alpha	2.001	1.4.E-04
